# Warming disrupts infection patterns and primarily accelerates *Ostreococcus*-virus infection dynamics in the Baltic Sea

**DOI:** 10.1093/ismeco/ycag227

**Published:** 2026-08-06

**Authors:** Carina Peters, C-Elisa Schaum, Luisa Listmann

**Affiliations:** Institute of Marine Ecosystem and Fisheries Science, University of Hamburg, Olbersweg 24, 22767 Hamburg, Germany; Institute of Marine Ecosystem and Fisheries Science, University of Hamburg, Olbersweg 24, 22767 Hamburg, Germany; Institute of Marine Ecosystem and Fisheries Science, University of Hamburg, Olbersweg 24, 22767 Hamburg, Germany

**Keywords:** *Ostreococcus,* virus, network dynamics, warming, pico-phytoplankton, Baltic Sea

## Abstract

Warming affects phytoplankton host–virus dynamics, but only a few model systems in a handful of ecosystems have been experimentally explored. Viruses influence biogeochemical cycling, phytoplankton population dynamics, and drive coevolution and adaptation. Assessing how host–virus interactions are affected by changing environmental conditions is crucial for understanding and predicting shifts in phytoplankton community structure and function. We first characterized general infection network dynamics through a comprehensive cross-infection experiment involving 31 *Ostreococcus* strains and 83 viruses, predominantly from the Baltic Sea. We found a modular network structure, characterized by generalist and specialist infections, coevolutionary history and phylogeny. To assess the impact of warming on infection dynamics, we then performed cross-infections with a representative subset of 12 selected host strains and 17 virus lysates per host each at 20°C, 22°C, and 24°C, temperatures generally below the optimal growth temperature for most hosts. We measured spot-plaque growth rates by tracking the timing and growth dynamics of spot-plaque formation through daily photos. Increasing temperature significantly affected susceptibility, spot-plaque formation, and growth, generally increasing rates but with variable responses among host–virus pairs, especially among generalist viruses. Such differences, often decoupled from host growth, indicate that warming adds more complexity to infection dynamics and further increases unpredictability of infection patterns. These results suggest that a shift in infection dynamics could potentially alter the role of the viral shunt in future oceans, reshape infection networks, and therefore affect biogeochemical cycling and food webs.

## Introduction

Microbial communities form the base of the food web, and phytoplankton–virus interactions are among the primary drivers influencing carbon and nutrient cycling. Through mechanisms such as the viral shunt or viral shuttle, they contribute to nutrient recycling and export of organic matter to the deeper ocean [1]. Phytoplankton–virus research has advanced considerably in understanding infection mechanisms [2–5], genomic diversity [6–9], and biogeochemical impacts [10–12]. However, studies characterizing and assessing natural phytoplankton–virus networks and their responses to environmental factors [13–15] remain scarce.

Infection outcomes can be highly specific to the selected host–virus pair [16], due to different virus infection phenotypes (generalist, specialist) and viral traits such as adsorption rate, latent period, and burst size [17, 18]. Host–virus pairs with a history of coevolution often exhibit different infection dynamics compared to ‘foreign’ combinations [19, 20], and these dynamics are further modulated by environmental conditions such as light and temperature [14, 21, 22]. There are only a limited number of studies that have addressed how host–virus dynamics and virus traits are affected by warming, and the results are variable depending on the host and virus combinations. For instance, warming can result in prevention of progeny viral production, can lead to virion production without any apparent cell lysis [13], or even lead to viral resistance [13, 23]. Increased temperature can also shorten latent periods and increase burst sizes [13, 17]. Despite these findings, comprehensive studies that span both a broad range of environments and multiple host–virus combinations within each environment are lacking, largely due to the immense experimental effort. While host diversity and variability in regard to temperature is increasingly being addressed over broader ranges [24–26], similar efforts focusing on virus–host dynamics and diversity therein remain limited.

The Baltic Sea is among the regions most projected to be affected by climate-driven warming. It is particularly sensitive to warming due to its semi-enclosed geography, shallow basins, and infrequent inflow from the North Sea. Between 1990 and 2018, the average sea surface temperature (SST) increased by +0.59°C per decade, with 2018 recording the highest SSTs since 1990 [27–29]. While mean Baltic Sea surface temperatures of the last decade are around 18°C in summer months [29], more recent projections predict an increase in frequency and length of marine heatwaves in the Baltic Sea [30], with past heatwaves (2018, 2021) reaching up to 25°C in coastal regions [31, 32]. Picoeukaryotes, including *Ostreococcus*, contribute to carbon cycling year-round, largely due to their proficiency in oligotrophic environments [33, 34]. But they also contribute significantly to the carbon cycle in eutrophic regions, like the Baltic Sea [35–37]. *Ostreococcus* and its associated viruses are distributed across a range of salinities throughout the Baltic Sea, from the Kattegat to the Bothnian Bay, with viral abundances generally higher in regions of elevated salinity [38, 39]. In addition, *Ostreococcus* is a tractable model organism for plaque-based infection studies, owing to its ease of cultivation and growth on solid media. *Ostreococcus* viruses are double-stranded DNA viruses (prasinovirus) [40] that play an important role in regulating host abundances, as they typically lyse the host cell [41]. However, some instances of infection without any apparent host cell lysis [42] or small susceptible subpopulations have also been reported [43].

In this study, we investigated the infection network dynamics among 31 *Ostreococcus* strains and 83 virus isolates originating from the North Sea, Kattegat, and Southern Baltic Sea. Using a cross-infection, spot-plaque assay approach, we examined a total of 2573 unique host–virus combinations at 18°C. We then explored the influence of warming on infection dynamics using a subset of hosts and viruses, quantifying susceptibility, time until visible infection, and spot-plaque growth rates at different temperatures, mostly below host thermal optimum for maximum growth rate (host thermal optima were determined in Peters et al. [24]). This enabled us to assess the thermal sensitivity of interactions and infection outcomes between different viruses, hosts, and origins. This approach allows for a scalable and comprehensive exploration of host–virus dynamics. While it only provides proxies for parameters such as lag time and infection rates, as viral parameters cannot be quantified, it provides a high-throughput and comparative framework to assess infection networks and infection outcomes across many host–virus combinations simultaneously—something that is rarely feasible with traditional one-host-one-virus experiments. Our results contribute valuable insights into the potential impacts of climate-driven warming on the viral infection network of the Baltic *Ostreococcus*-virus system and presents a new approach on investigating a broad range of host–virus combinations in changing environments.

## Materials and methods

### *Ostreococcus* and *Ostreococcus* virus isolation and sequencing

**Figure 1:**
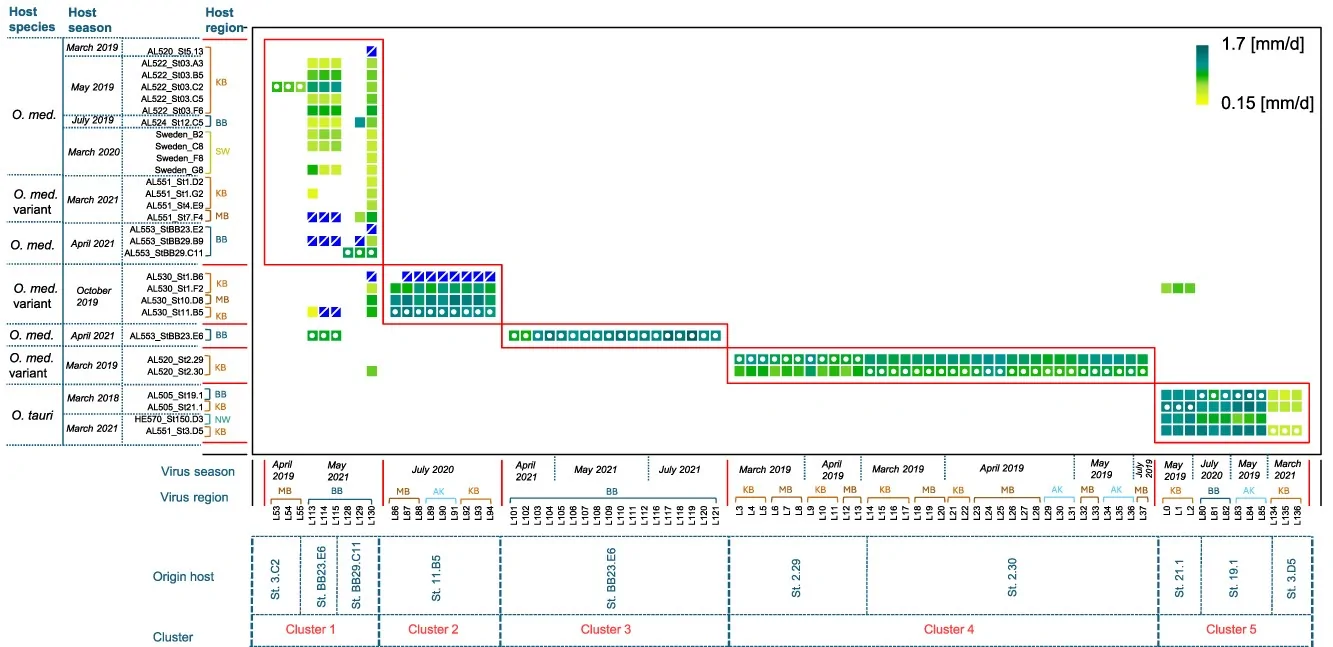
Results of cluster analysis based on binary data of infection. Hue indicates the spot-plaque increase rate. Squares with diagonal slashes indicate that no infection rate is available due to the infections being very late, and therefore not enough data points for rate analysis being available or the spot-plaques not showing clear borders for reliable size measurements. Lightest (yellow) hues correspond to slowest rates, and darkest (teal) hues correspond to fastest rates. Squares with dots in the middle indicate origin host–virus pairings. Empty (white) space indicates that the infection of that combination was unsuccessful. All host–virus combinations were tested; however, two host strains (AL530_St1.B4 and Sweden_B5) were not infected by any viruses and are therefore not represented in the matrix, as the clustering approach requires at least one positive interaction per strain to assign it to a cluster. On the *x*-axis are the viruses with virus isolation season, region, origin host, and cluster. On the *y*-axis is the infected host with their species, isolation season, and region. Region abbreviations: AK = Arkona Basin, BB = Bornholm, KB = Kiel Bight, SW = Sweden, MB = Mecklenburg Bight, NW = Norway.

The collection of hosts and viruses comprises 31 *Ostreococcus* hosts and 83 *Ostreococcus* viruses, which were isolated from 9 of the 31 hosts. Many of the viruses isolated from the same host share a similar host range and molecular identity, which is why they are often referred to in pairs of three, e.g. L113-L115 (Fig. 1, Supplementary Fig. S1). The *Ostreococcus* hosts [24, 39, 44] and virus strains [39] stem from the Southern Baltic Sea, Skagerrak, and North Sea (see Supplementary Tables S1 and S2 for more information about hosts and viruses). Most host names consist of a cruise and strain identifier, e.g. AL520_St5.13, where the ‘AL520’ identifies the cruise where water samples were collected (information about cruises can be found at The Federal Maritime and Hydrographic Agency of Germany) and ‘St5.13’ is the strain identifier. All hosts and viruses have also been cloned from a single cell or virus plaque and sequenced (18S rRNA and ITS-region for hosts [24], polymerase B (polB) for viruses) for species identification prior to the present study, following the same methods as outlined in Listmann et al. [39].

The host strains were previously identified as strains belonging to *Ostreococcus tauri* (*O. tauri)* and *Ostreococcus mediterraneus (O. mediterraneus)*, as well as a third strain group, designated as a more divergent *O. mediterraneus* variant [24]. This separation is based on 18S rRNA sequences, ITS-region sequencing, and unpublished whole-genome sequencing data (more detailed information on this designation can be found in [24]). Virus sequences were trimmed and cleaned up in Codon Code Aligner. Forward sequences (269 bp for polB) were used for phylogenetic tree building of polB virus sequences. Phylogenetic trees were inferred in R (Version 4.5.0) using the phangorn package (Version 2.12.1). The best-fit model (K80 + G(4)) was selected using BIC, and a maximum likelihood tree was generated with 1000 bootstrap replicates for node support using the ‘bootstrap.pml()’ function. Virus lysates (L103, L106, L107, L108) failed polymerase chain reaction (PCR) amplification and are therefore excluded in tree building.

### Cross infection on agar plates

First, an extensive cross-infection experiment was performed using all *Ostreococcus* strains (*n* = 31) and viruses (*n* = 83), resulting in 2573 unique host–virus combinations. Of these, 1176 correspond to previously tested combinations [39], which were included to assess reproducibility (which broadly gave the same results) as well as ensure standardized data collection across all samples. The remaining 1397 combinations represent novel host–virus pairings (due to 10 new hosts, 27 new viruses that were more recently isolated; some previously used viruses were lost) that were not available in the previous study. Host cultures were grown at 18°C (12:12 h light:dark, ~90–150 μmol m^−2^ s^−1^) until visibly green and subsequently diluted to 10^7^ cells mL^−1^. This temperature was chosen as the baseline, as it is the common garden temperature we maintain our *Ostreococcus* strains at and is the mean SST in summer months for the Baltic Sea [29]. Viral lysates were fixed in 0.5% glutaraldehyde, shock-frozen at −80°C, and later quantified via flow cytometry [45, 46]. Lysates were diluted to ~2 × 10^7^ viruses mL^−1^ to achieve a comparable inoculation density across the experiments.

**Figure 2:**
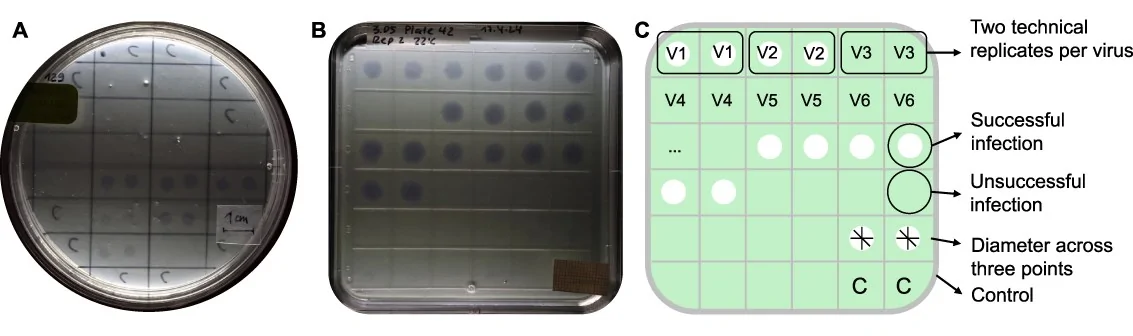
(A) Example of an agar plate from the large cross infection at 18°C (Host strain: HE570_St150.D3). (B) Example of an agar plate from the cross infection at different temperatures (Host strain: AL551_St3.D5). (C) Schematic of agar plate analysis and measurement of spot-plaques on photos.

*Ostreococcus* cultures were plated in petri dishes (0.1% final agar concentration), and lysates were spotted (2 μl) in duplicates onto the solidified culture in a grid layout. Control spots received no lysate (Fig. 2A–C). Plates were incubated under controlled light conditions (~50–150 μmol m^−2^ s^−1^) at their respective temperature treatments and photographed daily. Spot-plaque diameters were measured in ImageJ as a mean of orthogonal measurements (Fig. 2C; see Supplementary Methods for more detailed procedures). In addition, the day of first visible infection was tracked (DoI).

### Cross infections under warming

A subset of 12 of the 31 host strains was selected to perform cross infections under warming. These strains were selected to best represent the different regions, species, infection dynamics, as well as different optimum growth temperatures of the hosts (Supplementary Fig. S2). Each host was spotted with 17 different viruses (Supplementary Fig. S3 shows which host was infected with which viruses). Host strains were acclimated to the experimental temperatures 20°C, 22°C, and 24°C in triplicates in liquid cultures for 9.5–11 generations (12:12 h light–dark cycle, ~70–135 μmol m^−2^ s^−1^) with a starting concentration of 10^4^ cells mL^−1^. Temperatures were chosen so that carrying capacity would still allow cultures to grow visibly green in order to see plaque formation. At 8–9 generations, samples were also analysed for reactive oxygen species (ROS) production (Supplementary Fig. S4) to compare the initial stress response to the temperature between host strains. Once the samples were acclimated, they were diluted to 8 × 10^6^ cells mL^−1^, and agar plates were prepared in duplicates as described above. One strain did not survive at 24°C and was excluded. Plates showing initial host death followed by heterogeneous regrowth were omitted to ensure consistency (see Supplementary Methods for more details).

### Network analysis

We performed a bipartite network analysis using binary infection data (infected vs. not infected) to assess the modularity of the infection network. This was done in R using the ‘computeMod()’ function of the bipartite package (Version 2.20) [47–49]. To test for significance of observed modularity, we generated 1000 null model networks using the ‘r2d’ null model and calculated Q for each null network. Observed Q was then compared to the distribution of null Q-values, and z-scores were computed as follows:


$$ {\boldsymbol{z}}_{\boldsymbol{Q}}=\frac{{\boldsymbol{Q}}_{\boldsymbol{observed}}-{\overline{\boldsymbol{Q}}}_{\boldsymbol{null}}}{{\boldsymbol{\sigma}}_{{\boldsymbol{Q}}_{\boldsymbol{null}}}} $$

### Fitting and extraction of spot-plaque growth rates

Spot-plaque growth rates were estimated using linear regression of mean spot-plaque diameter of the technical replicates against day post infection (Supplementary Figs S5–S20), grouped by virus and infected host. Only models with an R^2^ > 0.7 were included into further analysis. Day of visible infection (DoI) is defined as the first day after infection that spot-plaques were visible. The Wilcoxon rank-sum test was used for analysing differences between two pairs (e.g. differences between original isolation host or foreign host). Differences between more than two groups were analysed using ANOVA followed by Tukey’s HSD for multiple comparisons (when assumptions were met) or the Kruskal–Wallis test followed by the Dunn’s *post hoc* tests. For the warmed treatments, we had duplicate agar plates; therefore, two spot-plaque growth rates were fitted for each host–virus combination at 20–24°C.

### Statistical analysis of temperature effect

We observed that spot-plaque growth rates tended to be mostly consistent among viruses sharing the same origin host. Therefore, we fitted a generalized additive model (GAM) for all the viruses from the same origin host for each infected host across temperature. The model was fitted using the ‘gam()’ function from the mgcv package (Version 1.9-1) in R using a gaussian distribution, including a smooth term for temperature (k = 4). The basis dimension (k) was set to 4, corresponding to the number of unique data points available. Model comparisons with lower k values showed improved fit for k = 4 (lower Akaike Information Criterion (AIC)), and diagnostics indicated no need for further adjustment.

To assess the effect of temperature on infection outcome, we fitted a generalized linear mixed-effects model (GLMM) with a binomial error distribution and logit link function using the lme4 package (Version 1.1-35.3) in R. The response variable was infection outcome (successful infection = 1, no infection = 0). Temperature was included as a categorical fixed effect with four levels (18°C, 20°C, 22°C, and 24°C). Unclear infection outcomes were treated as NA. To account for non-independence of observations and to allow baseline infection probability to vary among combinations, we included a random intercept for each host–virus pair (Infected_Host:Virus). Model fit was assessed through inspection of residuals simulated via the DHARMa package (Version 0.4.6) to evaluate dispersion, zero inflation, and uniformity of residuals. Model-based marginal means and predicted probabilities across temperatures were estimated using the emmeans package (Version 1.10.2), and pairwise comparisons between temperatures were adjusted with the Tukey method.

## Results

The Baltic Sea *Ostreococcus*-virus system reveals a broad ecological range of infection strategies. Across the 31 *Ostreococcus* host strains and 83 co-occurring viruses, interactions ranged from highly specific to generalist (Fig. 1) with significant differences in infection dynamics, as measured by the rate of spot-plaque growth and the timing of initial infection. Furthermore, we found that the entire infection landscape is reshaped by temperature, with individual virus–host interactions showing complex and varying responses to warming.

### The spectrum of infection strategies

Network analysis resolved the host–virus interactions into five distinct clusters (Q = 0.7143, z-score = 22.39), which partition the different infection strategies. Two of the 31 host strains (AL530_St1.B4 and Sweden_B5) were also not infected by any virus, displaying a high resistance level. Infection outcomes were highly reproducible, with only 2.38% of repeat infections (this includes all tested combinations, positive and negative outcomes) differing from Listmann et al. 2023 [39].

The modular structure of the infection network (Fig. 1, red rectangles) was primarily driven by viral host range. Highly generalist viruses in cluster one (mainly L113-L115, L130; isolated from two *O. mediterraneus* hosts in the Bornholm Basin) together successfully infected 18 different host strains. These infections showed the slowest overall spot-plaque growth rates (Fig. 1, lightest hue in squares) and often produced faint, blurred spot-plaques with variable clearing between hosts (L113-L115 especially, Supplementary Fig. S21a). In contrast, cluster three contained highly specialized viruses exclusively infecting their origin host (Fig. 1), despite originating from the same host strain that also yielded three of the generalist viruses. Phylogenetic analysis (Supplementary Fig. S1) revealed high genetic distances between those two groups.

The remaining clusters were structured by host phylogeny and geography. Cluster five consisted entirely of the four *O. tauri* host strains, which were susceptible to a common set of viruses regardless of their different origin regions, indicating a species-specific infection pattern (Fig. 1). *O. tauri* viruses L134-L136 produced spot-plaques made up of many small plaques rather than one single expanding spot-plaque (Supplementary Fig. S21b) and did not infect the Norway *O. tauri* host (HE570_St150.D3). In contrast, clusters two and four were composed exclusively of *O. mediterraneus* variant hosts and were primarily shaped by geography and isolation time, with viruses also forming distinct separate clades in the phylogenetic tree (Supplementary Fig. S1). Overall, spot-plaques in clusters three to five mostly showed strong spot-plaque clearings, and clusters two and three exhibited some of the fastest spot-plaque growth rates (Supplementary Fig. S22), suggesting highly efficient local infection dynamics.

**Figure 3:**
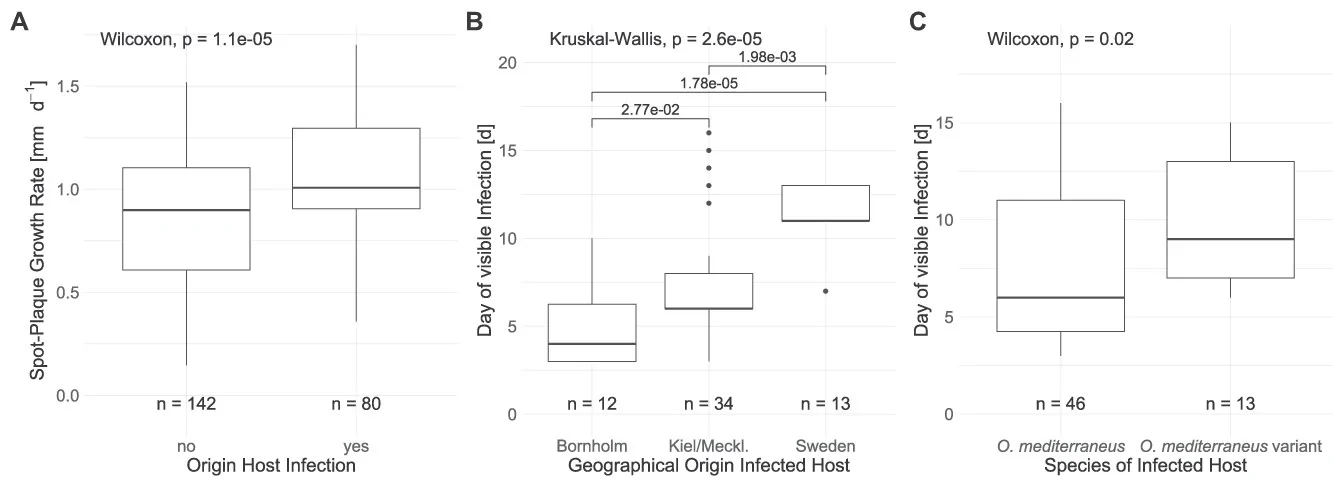
(A) Comparison of infection rates between viruses that infected their own host (yes), meaning the host they were isolated from or a foreign host (no). A Wilcoxon rank sum test was performed to test for statistical differences in rates (*P*-value = 1.1e-05). (B) Day of visible infection between regional origin of infected host (Kruskal–Wallis, *P* = 2.6e-05) and (C) species of infected host (Wilcoxon test, *P* = .02) for all infections with the highly generalist viruses L113, L114, L115, and L130. The boxes of the boxplots represent the 25th to 75th percentile of the data with the line within the box representing the median (50th percentile), the whiskers include the smallest and largest values without the outliers (outside 1.5 × IQR; shown as points).

Infection dynamics, measured as both spot-plaque growth rate and the DoI, varied across the network but were consistent within spot-plaque replicates. DoI was mostly consistent across clusters two to five but showed high variability in cluster one (Supplementary Figs S23 and S24), whereas spot-plaque growth rates varied between and within clusters (Supplementary Fig. S22). Spot-plaque growth rates and DoIs were significantly faster for original isolation hosts (squares with white dots in Fig. 1) compared to foreign hosts (Spot-plaque growth rate: Fig. 3A, Wilcoxon rank-sum test, W = 3661, *P*-value = 1.12e-05; DoI: Supplementary Fig. S25, Wilcoxon rank-sum test, W = 8815.5, *P* = 8.4e-12). For the highly generalist viruses (L113-L115, L130), DoI significantly differed between infected host origin regions (Kruskal–Wallis rank-sum test, χ^2^(2) = 21.11, *P* = 2.61e-09) and species (Wilcoxon rank sum test, W = 172, *P*-value = .02) with the smallest DoI exhibited by hosts matching the same origin region (Fig. 3B, Bornholm) and species (Fig. 3C, *O. mediterraneus*) as the virus and origin host.

Within cluster five, spot-plaque growth rates of viruses that infected all *O. tauri* hosts significantly differed between hosts (Spot-plaque growth rate: ANOVA: F_(3, 32)_ = 11.88, *P* = 2.17e-05), driven by significantly lower rates in Norway strain HE570_St150.D3 compared to Baltic *O. tauri* strains (Tukey’s HSD: all *P* > .009), which did not differ among themselves (Supplementary Fig. S26). Differences were most pronounced for the viruses from Bornholm and Arkona, the regions furthest away from the Norway station (Fig. 1). In addition, *O. tauri* viruses L134-L136 showed slower spot-plaque growth rates (Kruskal–Wallis rank sum test, χ^2^(2) = 21.22, *P* = 2.47e-05) and higher DoI (Kruskal–Wallis rank-sum test, χ^2^(2) = 40.004, *P* = 2.06e-09) across cluster five hosts (Supplementary Fig. S27), suggesting that not only host but also virus identity (here defined through origin host) drives infection dynamics.

### Temperature-driven reshaping of the infection network

The infection dynamics characterizing the network at the common garden 18°C treatment (Fig. 1) were profoundly altered by temperature changes across a narrow, suboptimal range [24] for the hosts (20–24°C). In general, warming predominantly accelerated infections, leading to faster spot-plaque growth and earlier onset of DoI. There was no variability for presence/absence or DoI of spot-plaque replicates; 93% of plate replicates showed identical DoI, the remaining replicates had a mean SE of 0.59 days. However, the responses of spot-plaque growth across temperature were far from uniform across host–virus pairs and susceptibility was also affected. Warming, therefore, added another factor of complexity to infection outcomes and infection dynamics.

**Figure 4:**
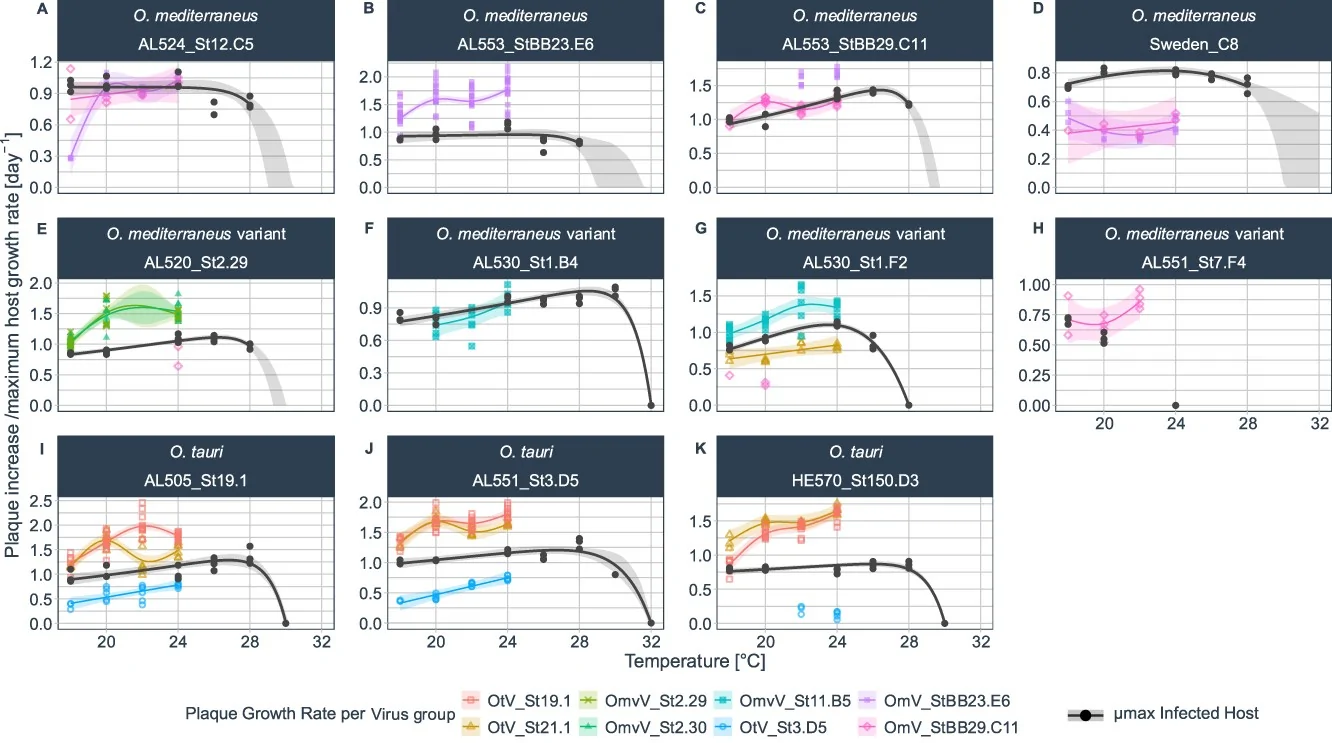
Fitted relationship between temperature and spot-plaque growth rate, estimated using a GAM. Shaded areas indicate 95% confidence intervals. A GAM was fitted for each virus group of viruses from the same origin host. Group names indicate virus clade based on origin host species and origin host identifier. OmV = *O. mediterraneus* virus, OmvV = *O. mediterraneus* variant virus, OtV = *O. tauri* virus. The different colours and shapes represent each of these groups, where number of viruses from the respective origin host can differ between the infected hosts (panels A–K). A smooth, nonlinear effect of temperature for each origin host (distinguished by shape and colour) within infected host (k = 4) was included. A gaussian distribution was used. Hosts thermal performance curves are added (closed circles) for better visualization of host growth dynamics at different temperatures without virus infection. Note that AL520_St2.29 was also infected by AL553_StBB29_C11 viruses at 20°C; however, limited measurements for those spot-plaques resulted in poor model fit for spot-plaque increase slopes at that temperature (R^2^ < 0.7).

Spot-plaque growth rates often shifted at every 2°C increment, whereas DoI typically changed only at the higher temperatures (22–24°C, Supplementary Fig. S3). Although the prevailing trend was an increase in growth rate with warming, thermal responses were highly idiosyncratic and frequently nonlinear (effective degrees of freedom (edf) of GAM-fits ranging from 1 to 2.96). Most groups had a significant temperature-dependent response (significant smooth for 14 out of 20 groups; all *P* < .02) and only few had weak or no temperature sensitivity (e.g. host Sweden_C8, Fig. 4D). The groups displayed distinct variability in plaque growth responses: rates increased with warming, some dropped with warming, and thermal responses of spot-plaques were both matched and mismatched with host growth.

Moreover, temperature acted as an ecological switch, determining whether an infection could occur at all. Out of the 187 tested host–virus pairs, 15 had changed infection outcomes (8.2%). The GLMM of the presence absence data revealed that infection success is significantly higher at 22–24° compared to 18–20°C (Tukey-adjusted pairwise comparisons: all *P* ≤ .009), with no significant differences within low (18 vs. 20°C, *P* = .97) or high (22 vs. 24°C, *P* = .95) temperature ranges. Infection probability peaked at 22°C, showing both colder and warmer treatments can affect susceptibility. Some infections that were successful at cooler temperatures disappeared entirely upon warming. Infected host AL530_St1.F2, which was infected by OmV_StBB29.C11 (Fig. 4G, pink open diamonds) at 18°C and 20°C, with spot-plaque growth rates already decreasing at 20°C showed an inhibition of infection at 22°C and 24°C. Conversely, some hosts became susceptible to infection only at warmer temperatures (e.g. Fig. 4C, AL553_StBB29.C11, purple closed squares). These contrasting outcomes indicate substantial thermal variability and nonlinearity in infection success across different host–virus pairs. However, the random effect of host–virus intercept was also very large (SD = 33.25), indicating the identity of host–virus pair is the dominant determinant of infection success.

Viruses that showed less diversity in the network (Fig. 1) also tended to be more similar in the response to warming. *O. tauri* hosts of the same regional origin showed comparable response shapes to viruses from certain clusters, e.g. the two Baltic *O. tauri* hosts (Fig. 4I and J, cluster five in Fig. 1) were susceptible to infection across all tested temperatures for the virus group OtV_St3.D5 (Fig. 4, blue open circles) and displayed similar responses to warming. The Norway *O. tauri* strain (Fig. 4K), which had already shown to be less similar to Baltic *O. tauri* hosts, diverged and was not infected by OtV_St3.D5 viruses at 18°C and 20°C and only became visibly susceptible with warming.

*O. mediterraneus* variant hosts often exhibited spot-plaque growth rates that closely matched their own growth performance (Fig. 4F–H), while responses among *O. tauri* hosts were more heterogeneous between hosts but also showed remarkable differences between the different viruses (e.g. Fig. 4I and K). For the *O. mediterraneus* variant host AL520_St2.29, the responses for the viruses from the two origin hosts in cluster four closely overlapped, in line with phylogenetic clustering, with rates steeply increasing toward 20°C and then plateauing, surpassing host maximum growth at the warmer treatments with more variation among viruses (Fig. 4E).

Lastly, we found prominent heterogeneity between the highly generalist viruses (Fig. 4, OtV_StBB23.E6 and OmV_StBB29.C11, pink open diamonds and purple filled squares) infecting different *O. mediterraneus* and *O. mediterraneus* variant hosts. This group also contained 9 of the 15 pairs with changed susceptibility. For example, OmV_StBB29.C11 infecting host AL524_St12.C5 (Fig. 4A) showed rates that increased linearly and more than doubled across the temperature range, while strain Sweden_C8 (Fig. 4D) only showed a slight non-significant linear increase with rates that were much lower than host growth. Contrary to that, AL553_StBB29.C11 (Fig. 4C) and AL551_St7.F4 (Fig. 4H) showed growth rates that appeared tightly coupled to host fitness, mirroring the host’s thermal performance curve. Host susceptibility in these viruses was also both affected at warm and cold temperatures. Together, these patterns highlight substantial variability in both infection dynamics and susceptibility across temperature in highly generalist viruses.

## Discussion

The Baltic Sea *Ostreococcus*-virus system is characterized by a mixture of specialist and generalist viruses, with infection patterns shaped by coevolutionary history and viral phylogeny. This diversity of infection strategies resulted in contrasting responses to warming, highlighting that temperature effects depend on the underlying host–virus interaction. While generalist infections tended to show more variable outcomes under warming, specialist infections often responded more consistently when they shared similar baseline infection characteristics. Together, these findings demonstrate that understanding infection network structure at current day temperature is essential for interpreting and understanding host–virus dynamics under warming conditions. Accordingly, we first discuss the baseline infection network before considering how warming influenced infection dynamics.

### The Baltic *Ostreococcus*-virus infection network

The Baltic *Ostreococcus*-virus infection network showed pronounced modularity, in contrast to the predominantly nested structure often reported for bacteriophage networks [50]. This modularity reflects a dominance of highly species- and strain-specific viruses alongside a small number of generalist viruses, with limited nestedness within modules, consistent with a Mediterranean *O. tauri-*virus system [51]. Our results extend previous findings by showing that these generalist-specialist dynamics span multiple *Ostreococcus* species and are shaped not only by isolation time [39], but also geography, in line with geographically structured and often modular host–virus networks across marine systems [52, 53]. The alignment between viral phylogeny and network structure suggests that evolutionary constraints, rather than purely ecological context, limit shifts in host range, consistent with previous findings linking *Ostreococcus* infectivity to genetic similarity [39, 51]. However, some variation, such as between *O. tauri* viruses, could not be explained by polB similarity or geographic origin alone and would need more complex genomic analysis [51, 54].

The coexistence of specialist and generalist viruses suggests the Baltic *Ostreococcus* viruses employ multiple viral infection strategies, rather than one dominant mode. Consistent with previous works [50, 51, 55], generalist viruses in this study infected the most resistant hosts. High resistance of hosts often comes with a cost of resistance (COR), e.g. in fitness. However, we found no clear evidence of such costs, given the high variability in host growth rates within cluster one and relative to other clusters (at 18°C; Supplementary Fig. S28). This is consistent with studies in *O. tauri* showing no growth penalty of resistance in the absence of viruses [42, 56], although such costs may only emerge under competition [42]. The pronounced heterogeneity in spot-plaque growth rates and clearing among generalist infections suggests substantial variation in infection efficiency and host resistance. This may reflect host-mediated regulation of infection efficiency, as shown for *Bacteroidetes* viruses [57] or phenotypic plasticity of host susceptibility [23, 58]. Similar strain-specific variation has been documented for Baltic Sea bacteriophages and microalgal viruses, where infections may be incomplete or show reduced lysis, with host growth still possible [59, 60]. Accordingly, faint spot-plaques and low spot-plaque expansion rates likely reflect partial infectivity or reduced viral replication [61], whereas specialist infections are more consistent with efficient lysis. Similarly, spot-plaques made up of several small plaques could reflect cell-to-cell heterogeneity in resistance [62].

Spot-plaque growth rates were generally higher on original host strains, suggesting shared local adaptation and coevolution also influence infection efficiency [54, 63]. In *O. tauri*, however, viruses frequently cross-infected hosts from different regions and isolated up to 3 years apart, with only few differences in spot-plaque growth rates between hosts, indicating that infection-relevant traits might be sufficiently conserved to allow efficient infection beyond immediate co-occurring lineages. Furthermore, *O. tauri* viruses cross infected a single *O. mediterraneus* variant host, revealing rare cross-species infection.

Together, these diverse infection strategies, combined with host resistance heterogeneity, may contribute to maintaining virus and host strain-level diversity, thereby providing stable ecosystem function [53, 64]. Specialists persist through efficient infection of specific hosts, and generalists persist through broader but less efficient infection of many hosts [50, 65]. Such dynamics may be particularly relevant in fluctuating environments characterized by resistance heterogeneity and shifting susceptibility of populations [62, 66]. By enabling differential top-down control, such a strategy can contribute to the persistence of generalists [67], as specialization may not always ensure virus persistence in such environments [68]. However, as our experiments were limited to pairwise interactions, future work incorporating mixed-culture experiments involving both generalist and specialist viruses are necessary, which might also reveal a possible hidden COR mechanism of hosts through competition.

### Effect of warming

Our study shows that warming affected host–virus dynamics in a strongly host–virus-dependent manner. Although many combinations exhibited increased spot-plaque growth rates with warming, responses were often nonlinear, and both high and low temperatures altered susceptibility. These patterns broadly align with the thermal mismatch hypothesis, which predicts reduced infection success as environmental conditions diverge from host thermal optima [69]. For many *Ostreococcus* strains, warming moved conditions closer to host growth optima, potentially facilitating faster viral replication. Consistent with this, increased host growth has been linked to shorter latent periods and higher burst sizes, which could manifest as earlier days of infection and faster spot-plaque expansion, respectively [70, 71]. Similar patterns have been found for *Micromonas* and *Chaetoceros* infection dynamics in relation to temperature [13, 17, 72].

However, we also found the absence or weakening of spot-plaques at higher temperatures and changes in infection dynamics decoupled from host thermal performance. Other studies have shown high temperature resistance [13, 23] as well as increased complexity of infection outcomes nearing or reaching T_opt._ [13] with variable responses, not always reflecting host growth dynamics [17, 72]. In *Micromonas*, a close relative of *Ostreococcus*, temperature increases beyond T_opt_ can induce chronic infections with no detectable lysis or a switch to total resistance [13]. Chronic infection mechanisms have also been documented for *Ostreococcus* viruses [42], suggesting the absence or weakening of spot-plaques at higher temperatures could also reflect reduced lysis or altered viral life-history strategies that cannot be resolved with plaque-based methods.

In our study, temperature-dependent effects were particularly variable for the highly generalist viruses. Among more specialist viruses and viruses associated with host–virus clusters sharing a coevolutionary history, temperature responses were often more similar. Generalist viruses, which already exhibited high variability in infection dynamics within the baseline network, also showed the most variable responses to warming, including changes in spot-plaque growth rates, delays in infection, and inhibition of lysis even under favourable host growth conditions [24]. As generalist viruses tend to infect more resistant hosts with lower overall infection efficiency [61], temperature changes may disproportionately affect these interactions, thereby further increasing variability and reducing infections for more resistant hosts and potentially revealing trade-offs between host range breadth and thermal sensitivity. Because hosts originated from different regions and isolation periods, they likely shared little evolutionary history with the generalist viruses. In ectotherms, such limited host–virus coevolution has been linked to more severe changes in infection outcomes under warming [20]. Similarly, the host-dependent variability in *O. tauri* hosts from different regions suggests that co-occurrence history and geography interact with temperature to shape infection dynamics.

The differing degrees of overlap between infection dynamics and host growth, as well as ROS production, further suggest that viruses vary in their sensitivity to host physiology and that host cell state is not the primary driver of infection outcome. Zimmerman et al. [73] demonstrated that *Ostreococcus* viruses differ in their sensitivity to host physiology and that greater resilience to host performance incurs viral fitness trade-offs. Such trade-offs may help explain why infection dynamics of generalist viruses frequently diverged from host growth patterns in our study. However, we also see differing levels of overlap between the same virus infecting different hosts, indicating a strong interplay of sensitivity to host physiology, host resistance, temperature effects, and infection efficiency. Similar variability has been reported for *Micromonas polaris* systems, where temperature altered virus production but host–virus pairs differed markedly in burst size, latent period, infectivity, and coupling to host growth [17]. Such interactions are also likely responsible for fluctuations in the responses, leading to nonlinear changes in spot-plaque growth rates.

Despite inherent differences between infections on semi-solid agar and in liquid cultures, such as reduced diffusion, spatial immobilization of hosts, and localized, linear expansion patterns [74], the spot-plaque method captured complex, interaction-specific thermal responses comparable to those reported from liquid culture experiments. This suggests that plaque-based assays can provide valuable insight into how warming modulates infection dynamics, even if absolute rates differ between experimental systems. Overall, our results indicate that temperature acts as an ecological filter that alters infection dynamics by selectively enabling, suppressing, or modifying specific host–virus interactions and susceptibility rather than uniformly accelerating all infections. Furthermore, specialist and generalist viruses might be differently affected by warming, as warming may increase complexity more in generalist than specialist and coevolved infections. Such nonlinear and host- and virus-specific thermal responses imply that microbial community structure under warming may become increasingly variable and difficult to predict [14]. Because viruses play a central role in regulating phytoplankton populations [75], warming may shift microbial community composition and restructure host–virus networks. However, warming rarely occurs in isolation and is often accompanied by changes in nutrient availability [76] that further influences community composition and competitive outcomes among phytoplankton groups [77]. Under the combined influence of multiple environmental drivers, viral infections tightly coupled to host growth may therefore respond more strongly than those less dependent on host physiology, increasing the complexity and unpredictability of microbial ecosystem responses beyond temperature effects alone.

## Supplementary Material

SupportingInformationReworked_30072026_ycag227

## Data Availability

The data supporting the findings of this study are available in the Supporting Information. R codes and data sets used for statistical analysis are available on Zenodo (https://doi.org/10.5281/zenodo.18390728).
