# Supplementary material for: Warming disrupts infection patterns and primarily accelerates Ostreococcus-virus infection dynamics in the Baltic Sea
Source: ISME Commun. 2026 Aug 6;6(1):ycag227. doi: 10.1093/ismeco/ycag227 (PMC13518053; doi:10.1093/ismeco/ycag227)
Supplement: Supplementary file 1 [file supportinginformationreworked_30072026_ycag227.pdf]

Supporting Information for

Warming disrupts infection patterns and primarily accelerates *Ostreococcus*-virus infection dynamics in the Baltic Sea

Short title: Warming Alters Infection Dynamics

Carina Peters*[1], C.-Elisa Schaum[1], Luisa Listmann[1]

[1]Institute of Marine Ecosystem and Fisheries Science, University of Hamburg, Hamburg, Germany

*Corresponding author: Carina Peters

Email: carina.peters@uni-hamburg.de

This PDF file includes:

Figures S1 to S28

Tables S1 to S2

Supplementary Methods

Supplementary Figures

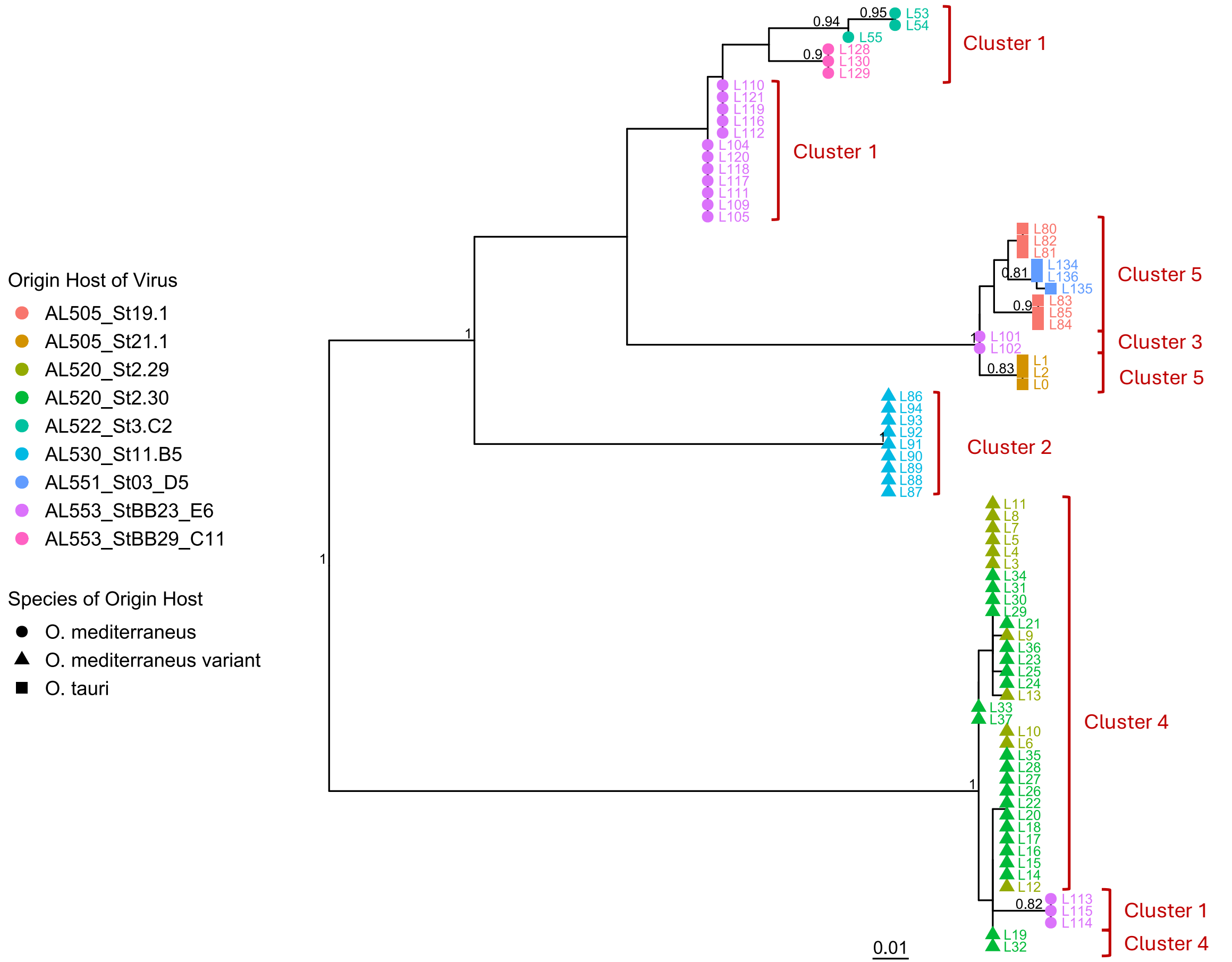

Supplementary Figure S1 Phylogenetic tree of virus polB sequences (269bp). The colours indicate the host strain the virus was isolated from. Lysate names consist of the short ID, isolation host (cruise + strain name, also highlighted by colour), isolation cruise and station of virus as well as region abbreviation (BB= Bornholm, MB=Mecklenburg Bay, KB= Kiel Bight, AK=Arkona Basin). The last number in the name indicates the virus number from that region/station combination as there is often more than one virus from the same water sample. Shape of node points indicate origin host species (circle = *O. mediterraneus* variant, triangle = *O. mediterraneus*, square = *O. tauri*).

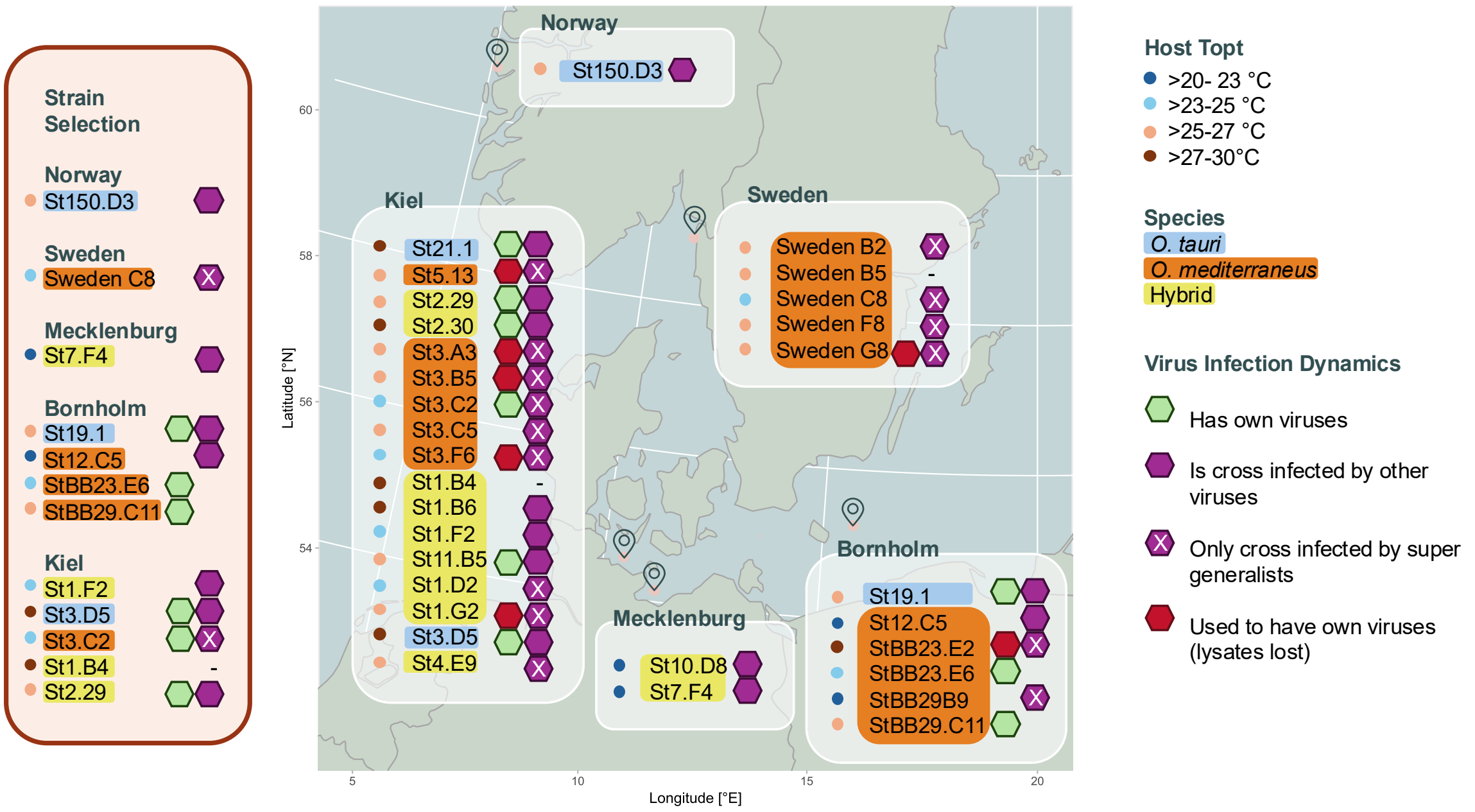

Figure S2 Illustration of host selection process for cross infection under warming. On the left is the final strain selection and on the right is an overview of all host strains used in the whole cross infection. The infection dynamics for each host are shown with hexagons, i.e. if it has (and therefore was infected by) own viruses (green hexagon), if it was cross infected by other viruses (purple hexagon), the special case of being cross infected by only the super generalist viruses (purple hexagon with cross) and lastly if it used to have viruses and is therefore known to be susceptible, but those viruses were lost (red hexagon). The hosts are marked by species with light blue for *O. tauri*, orange for *O. mediterraneus* and yellow for the hybrid type. In front of each host is a circle indicating the range its optimum growth temperature (T_{opt}) falls in, from dark blue (colder optimum growth temperatures) to dark red (for warmest T_{opt}). The boxes group the host strains by their different isolation locations.

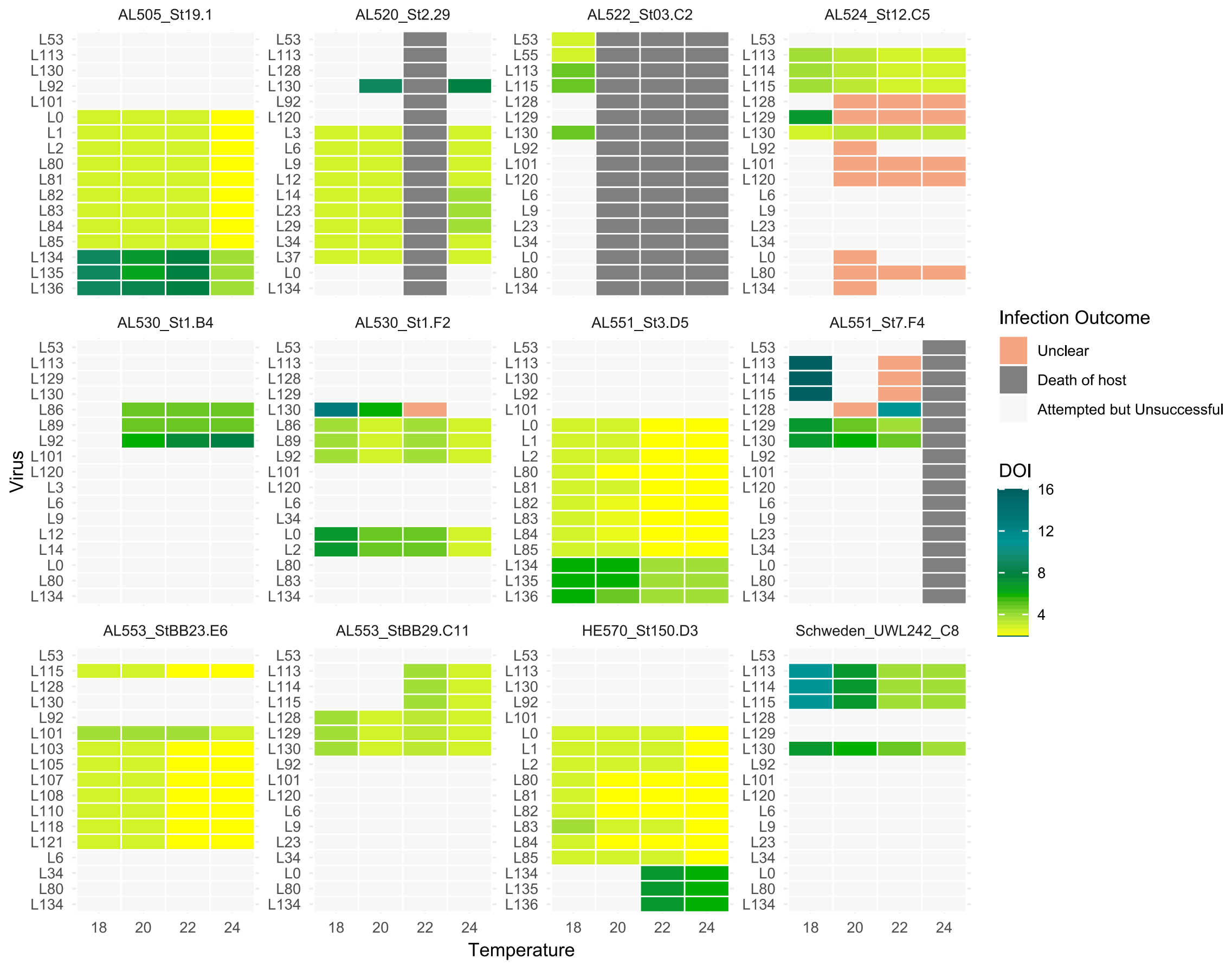

Figure S3 Overview of changes of first visible day of infection at the different experimental temperatures. Death of host refers to death in acclimation for strain AL551_St7.F4 and for strains AL520_St2.29 and AL522_St3.C2 it refers to after plating with molten agar, as cells exhibited transient depigmentation and reduced apparent viability. Unclear refers to infection outcomes that were not clearly identifiable, and death of host refers to either host cultures dying during acclimation or after agar pouring.

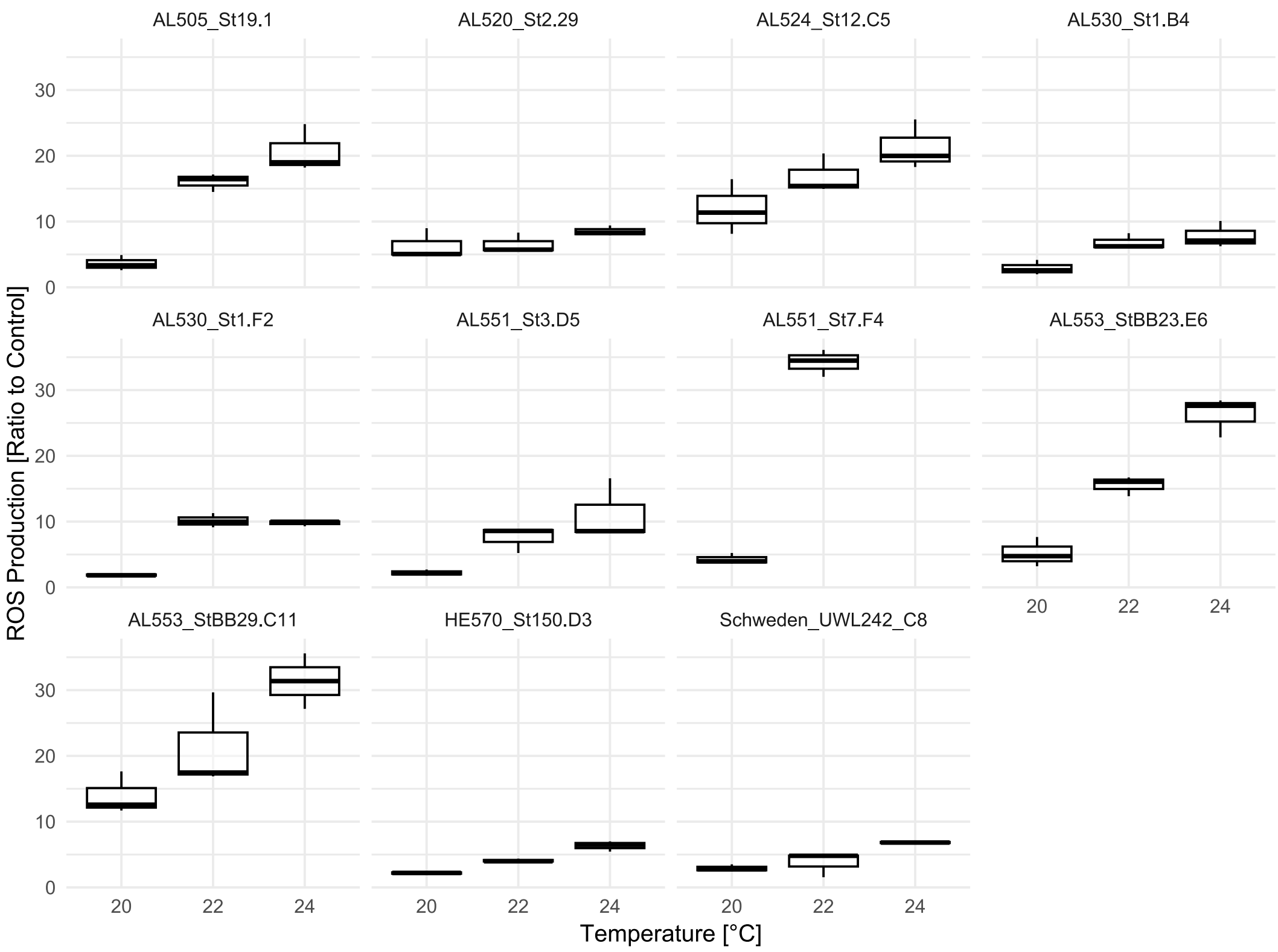

Figure S4 ROS production, displayed as the ratio of FITC fluorescence between ROS treated samples and the control sample. Time point of measurement was at 8-9 generations of acclimation to the according temperature (before infection with viruses). Samples were incubated for 15 hours before measurement. For most strains temperature increase caused an increase in ROS production, but intensity varied between strains.

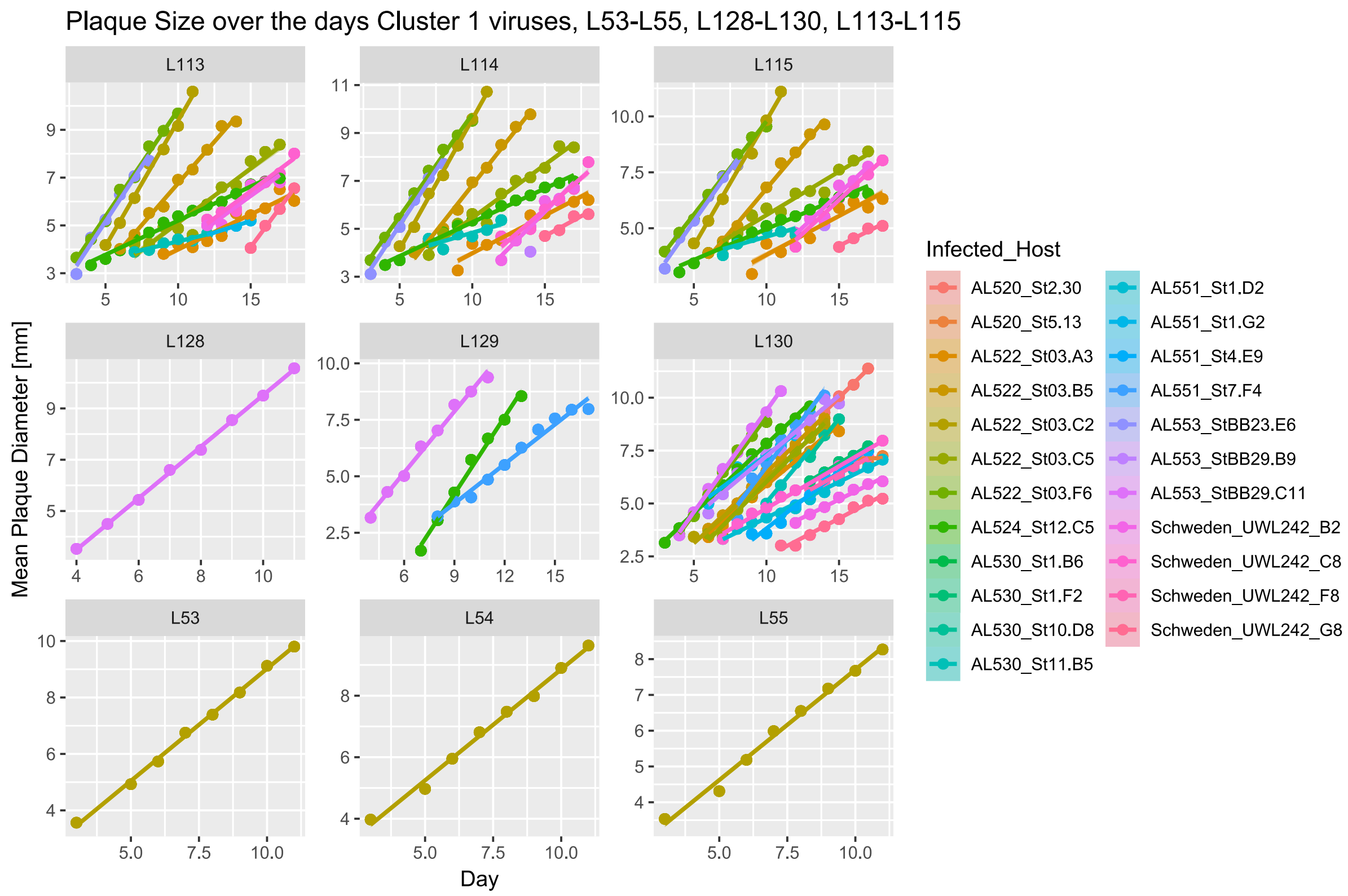

Figure S5 Slope fits of spot-plaque increase rates across days for infections in Cluster one. Shaded areas indicate standard errors from linear model fits. Colors indicate the different infected hosts per virus.

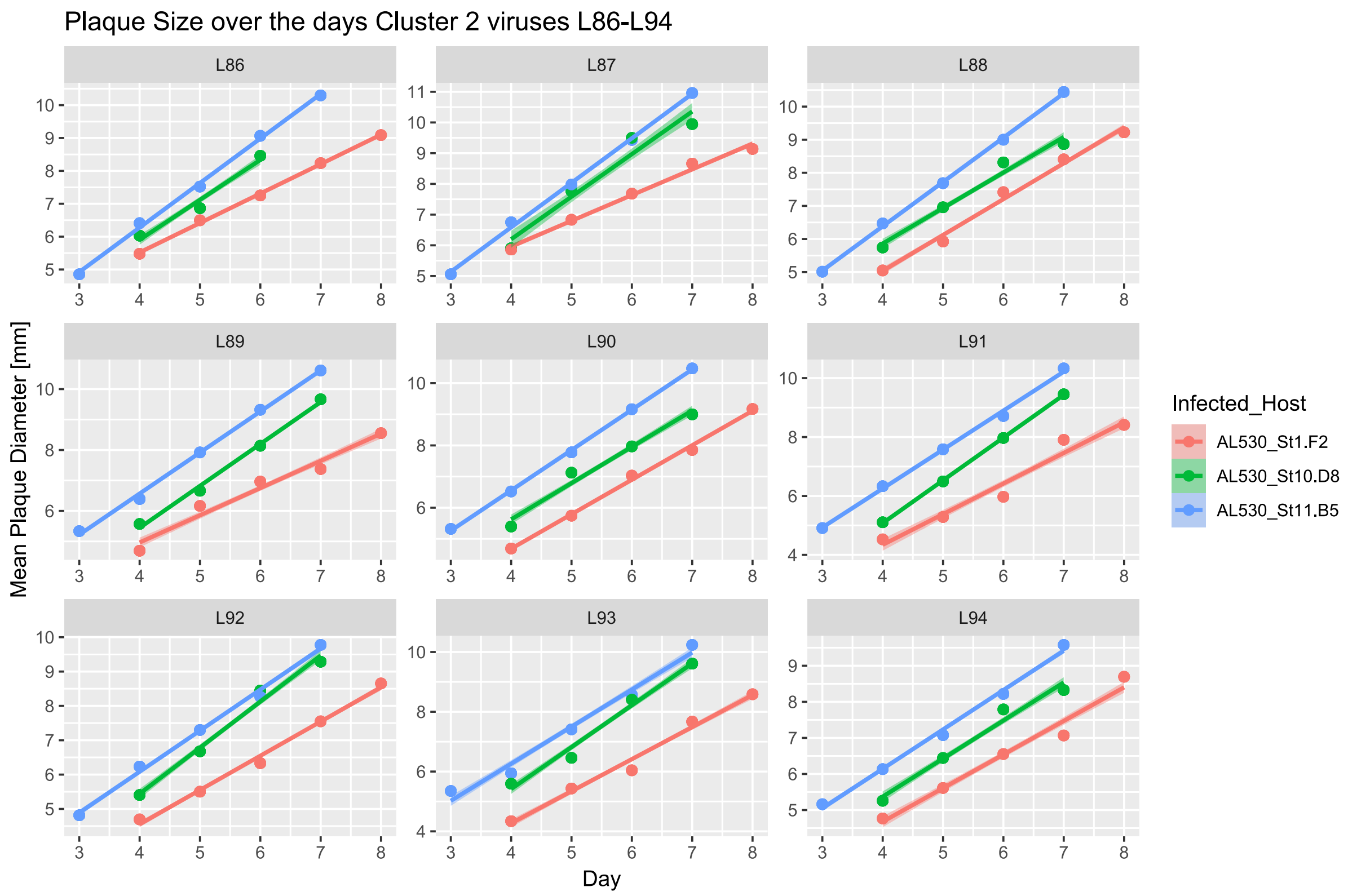

Figure S6 Slope fits of spot-plaque increase rates across days for infections in Cluster two. Shaded areas indicate standard errors from linear model fits. Colors indicate the different infected hosts per virus.

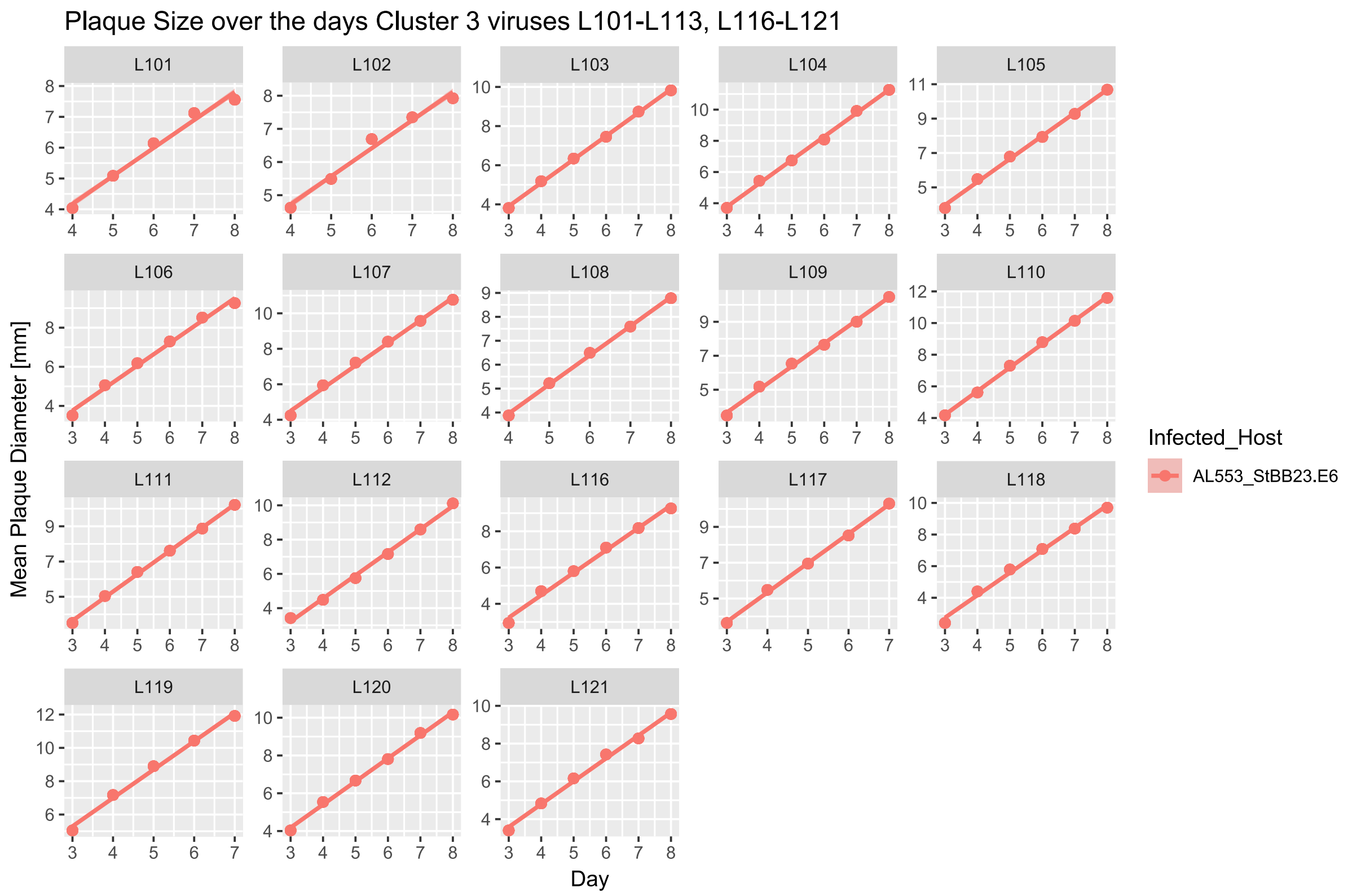

Figure S7 Slope fits of spot-plaque increase rates across days for infections in Cluster three . Shaded areas indicate standard errors from linear model fits. Colors indicate the different infected hosts per virus.

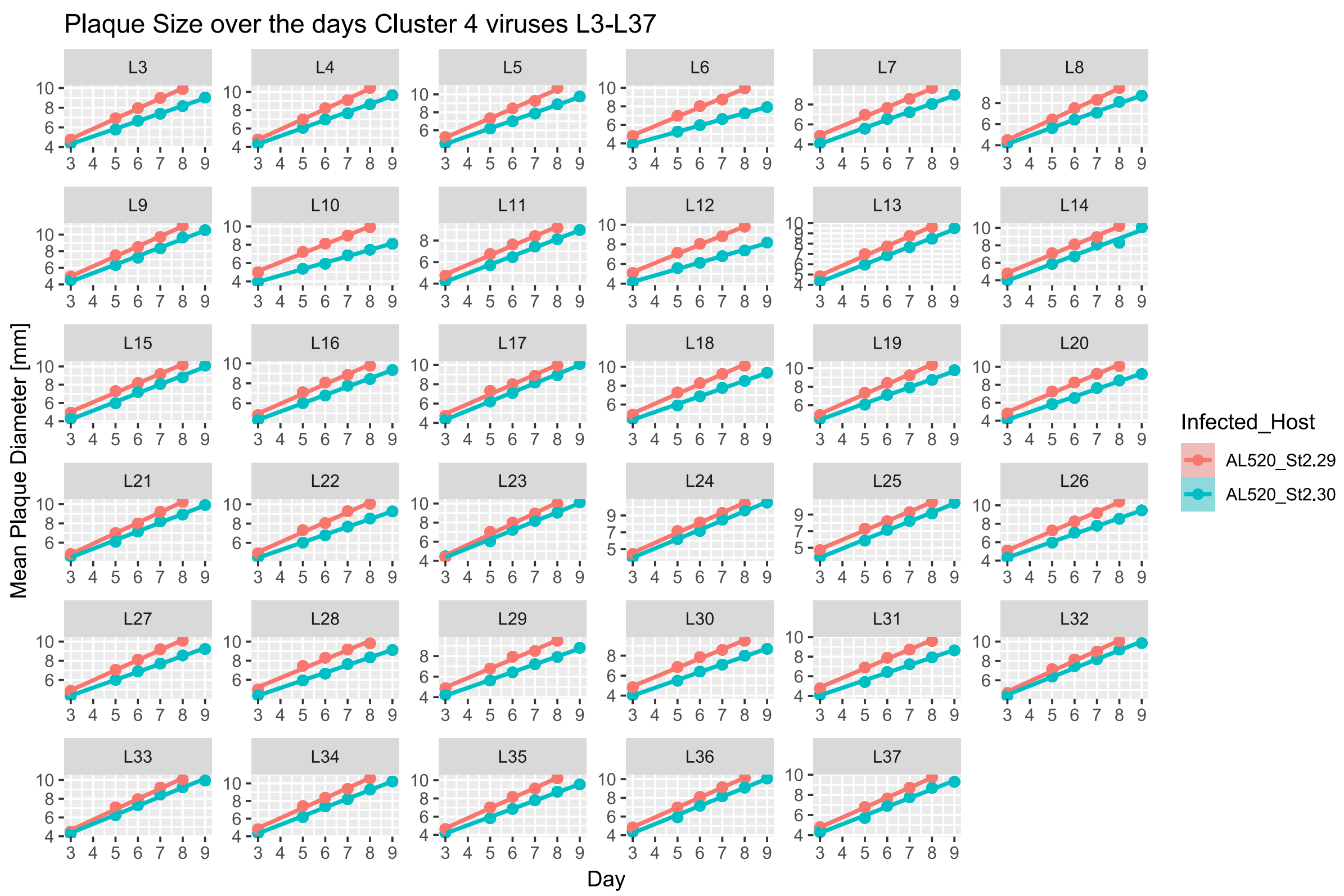

Figure S8 Slope fits of spot-plaque increase rates across days for infections in Cluster four. Shaded areas indicate standard errors from linear model fits. Colors indicate the different infected hosts per virus.

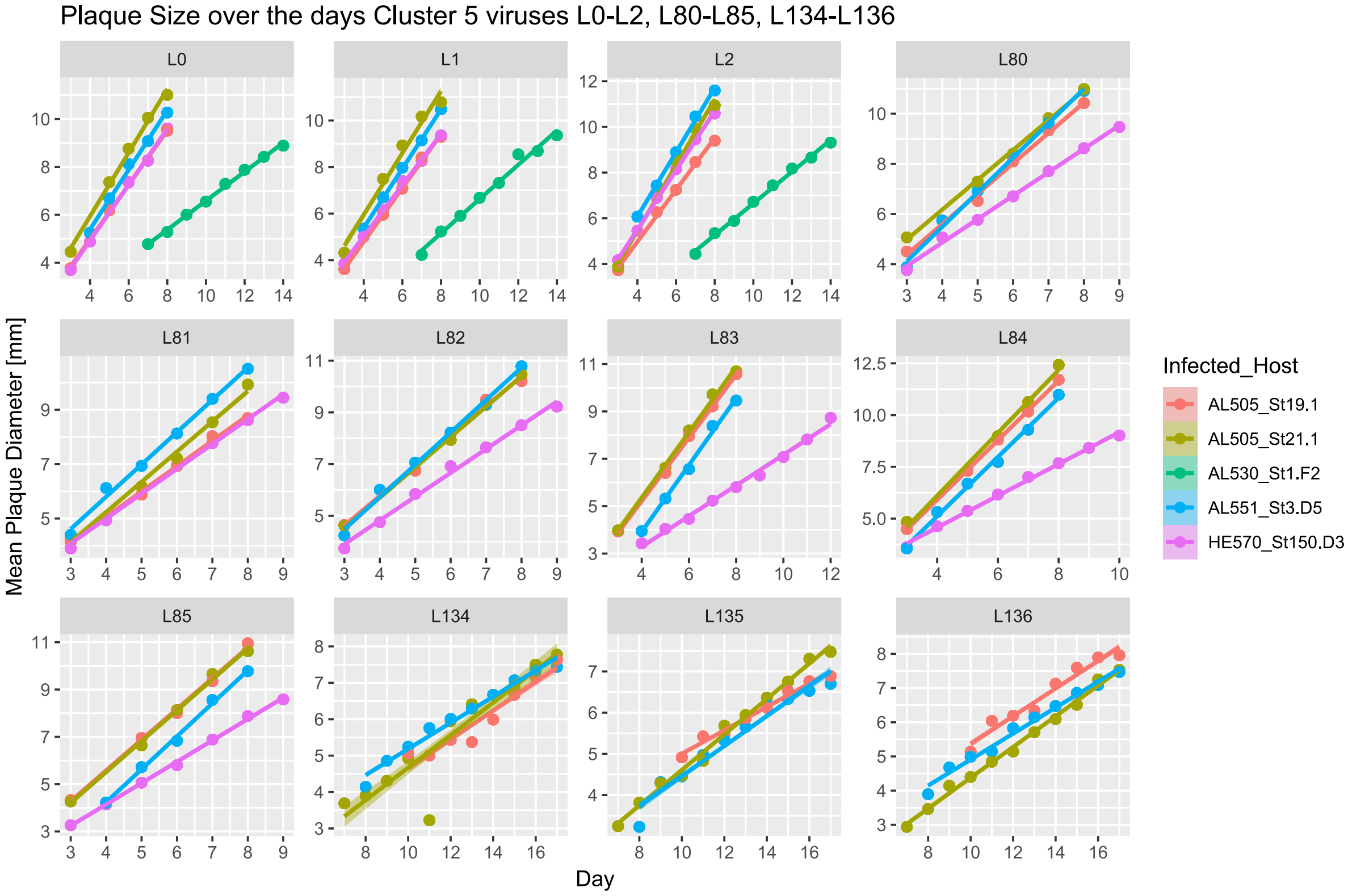

Figure S9 Slope fits of spot-plaque increase rates across days for infections in Cluster five. Shaded areas indicate standard errors from linear model fits. Colors indicate the different infected hosts per virus.

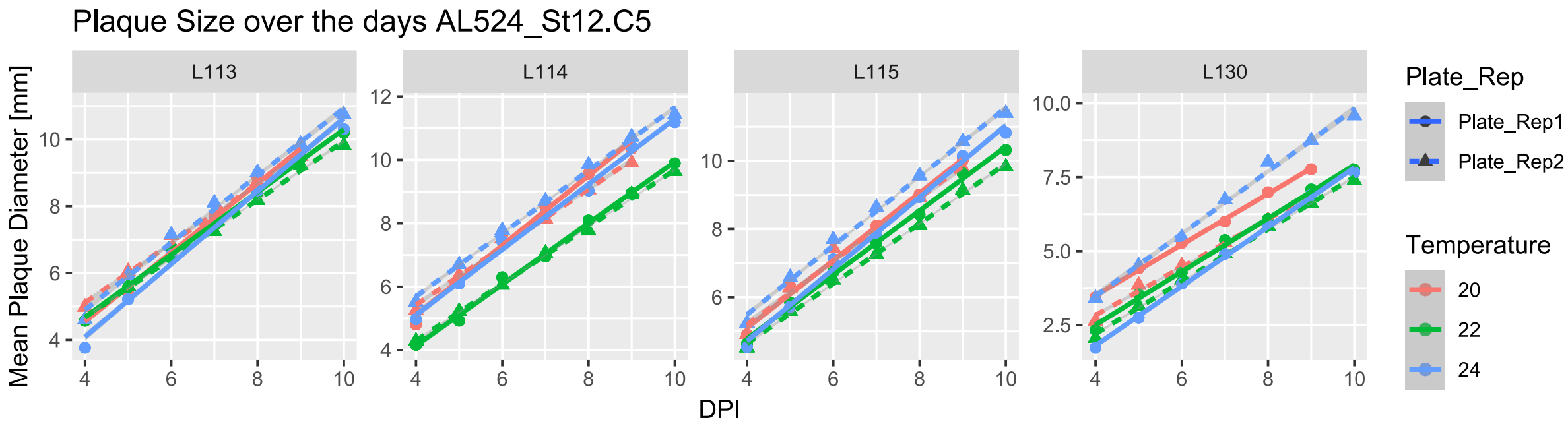

Figure S10 Slope fits of spot-plaque increase rates across days for infections for warmed treatments of host AL524_St12.C5. Shaded areas indicate standard errors from linear model fits. Shape indicates plaque replicate. Colors indicate the different temperatures.

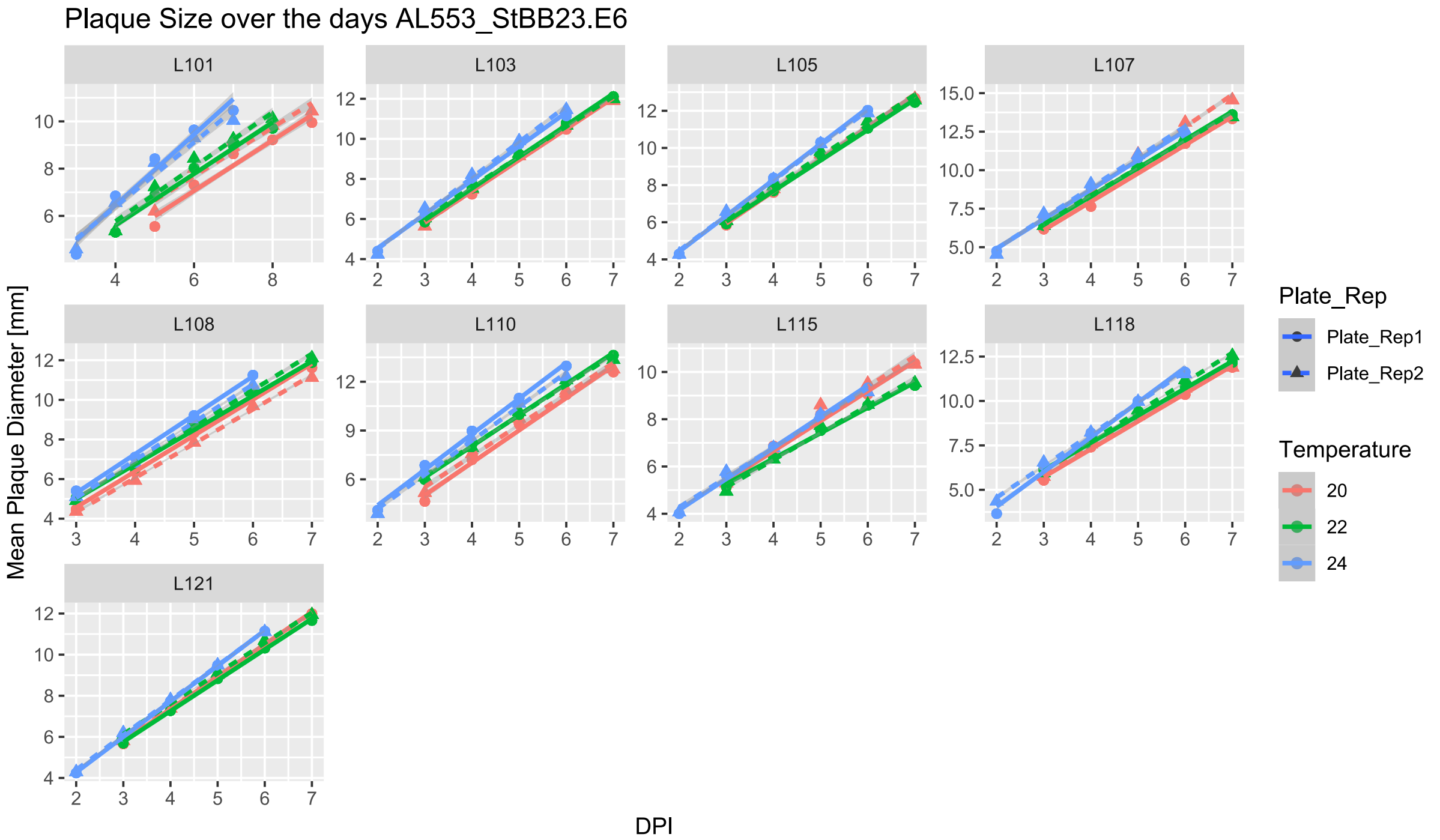

Figure S11 Slope fits of spot-plaque increase rates across days for infections for warmed treatments of host AL553_StBB23.E6. Shaded areas indicate standard errors from linear model fits. Shape indicates plaque replicate. Colors indicate the different temperatures.

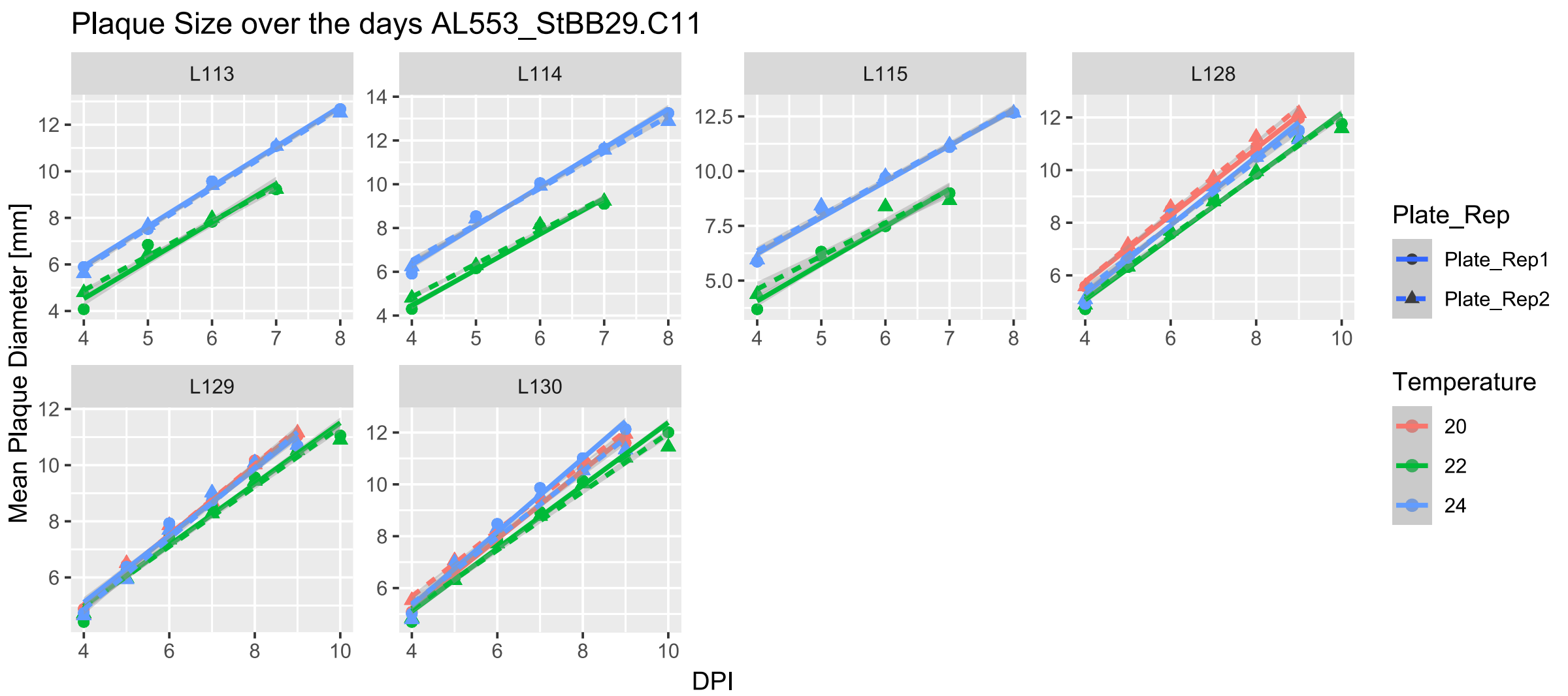

Figure S12 Slope fits of spot-plaque increase rates across days for infections for warmed treatments of host AL553_StBB29.C11. Shaded areas indicate standard errors from linear model fits. Shape indicates plaque replicate. Colors indicate the different temperatures.

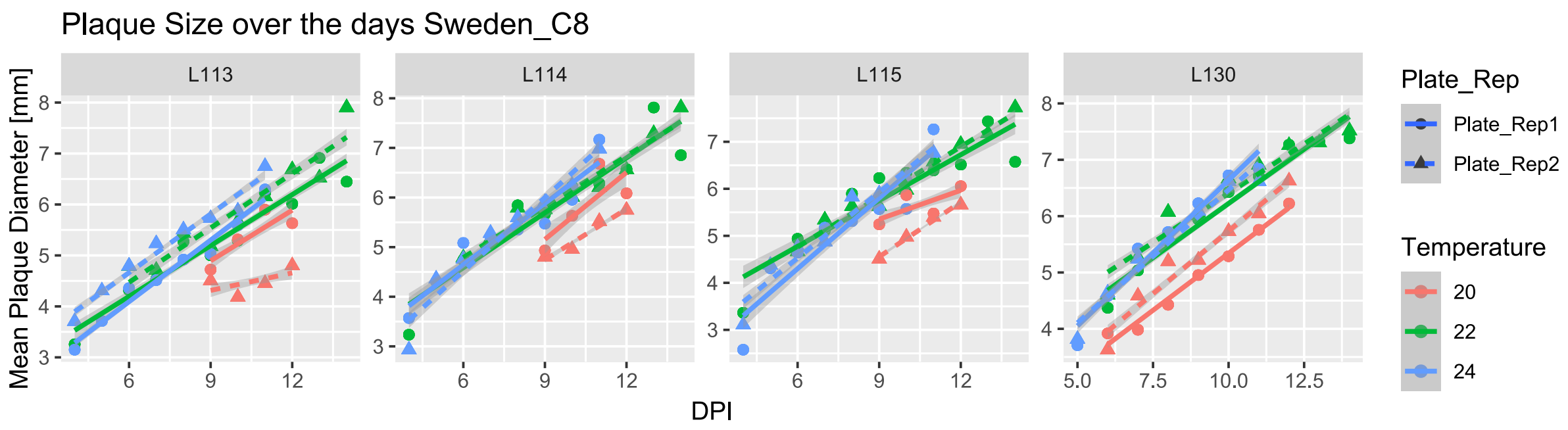

Figure S13 Slope fits of spot-plaque increase rates across days for infections for warmed treatments of host Sweden_C8. Shaded areas indicate standard errors from linear model fits. Shape indicates plaque replicate. Colors indicate the different temperatures.

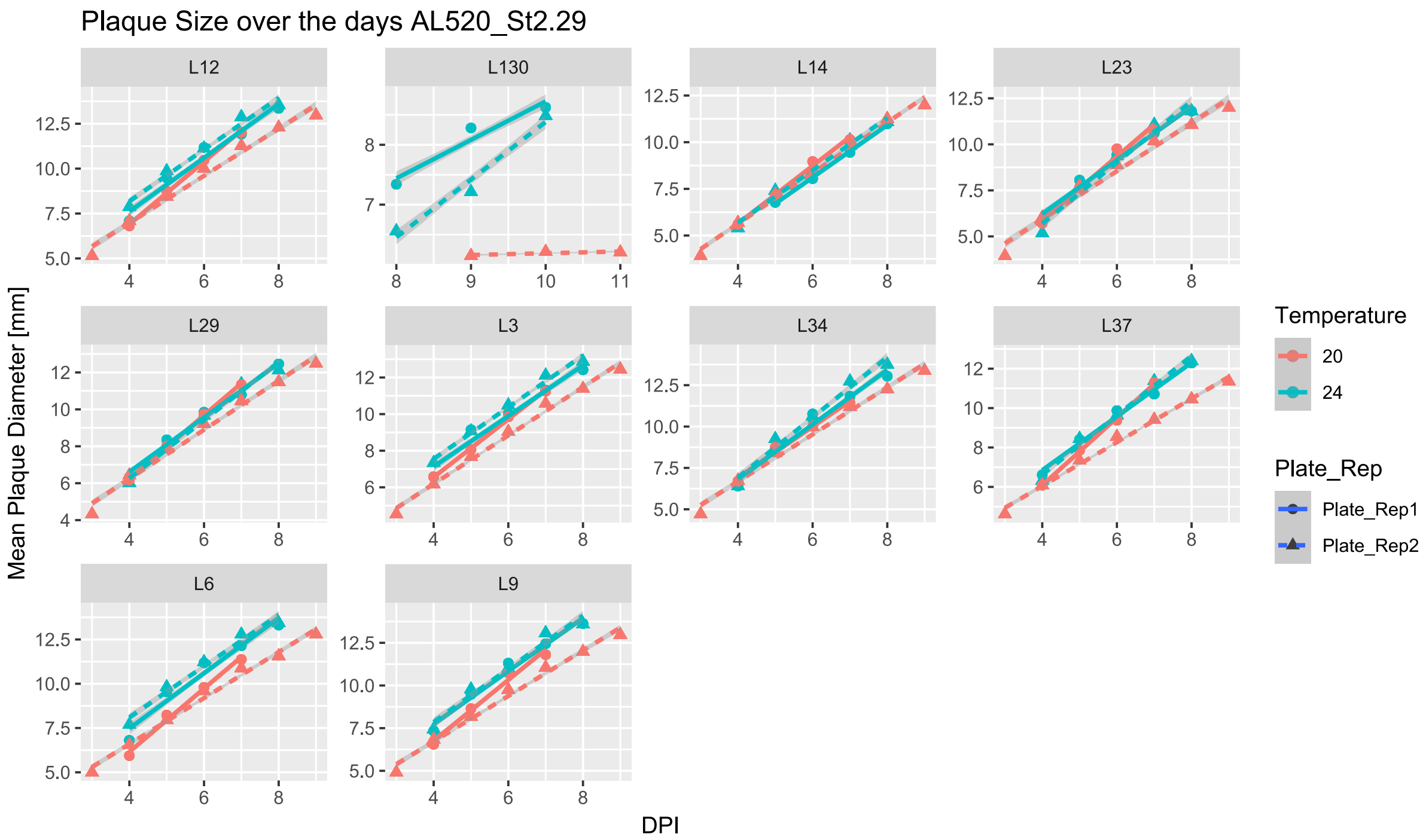

Figure S14 Slope fits of spot-plaque increase rates across days for infections for warmed treatments of host AL520_St2.29. Shaded areas indicate standard errors from linear model fits. Shape indicates plaque replicate. Colors indicate the different temperatures.

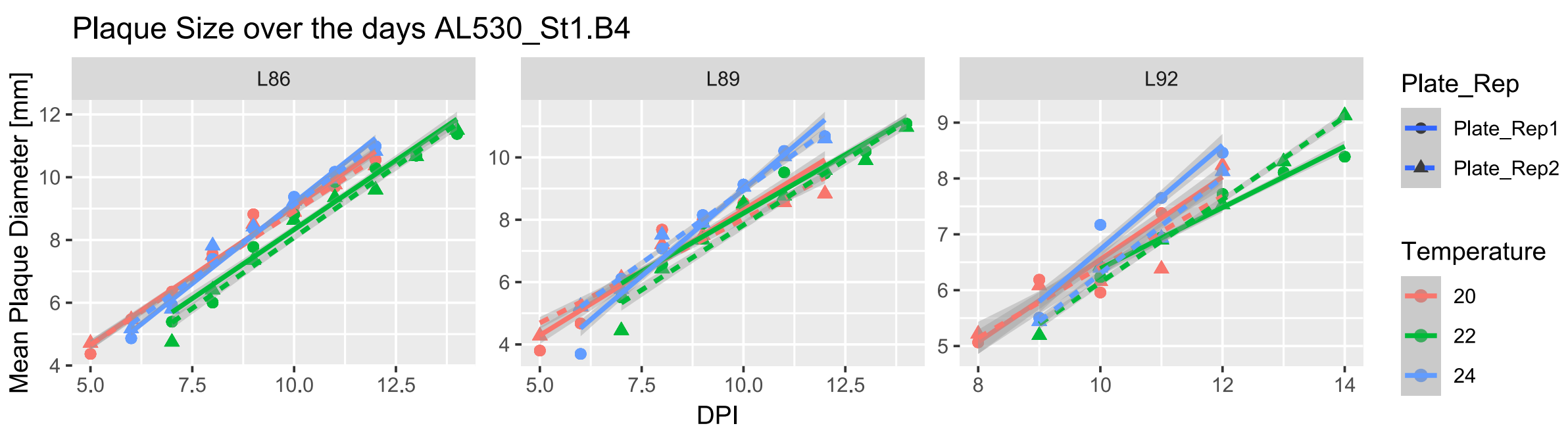

Figure S15 Slope fits of spot-plaque increase rates across days for infections for warmed treatments of host AL530_St1.B4. Shaded areas indicate standard errors from linear model fits. Shape indicates plaque replicate. Colors indicate the different temperatures.

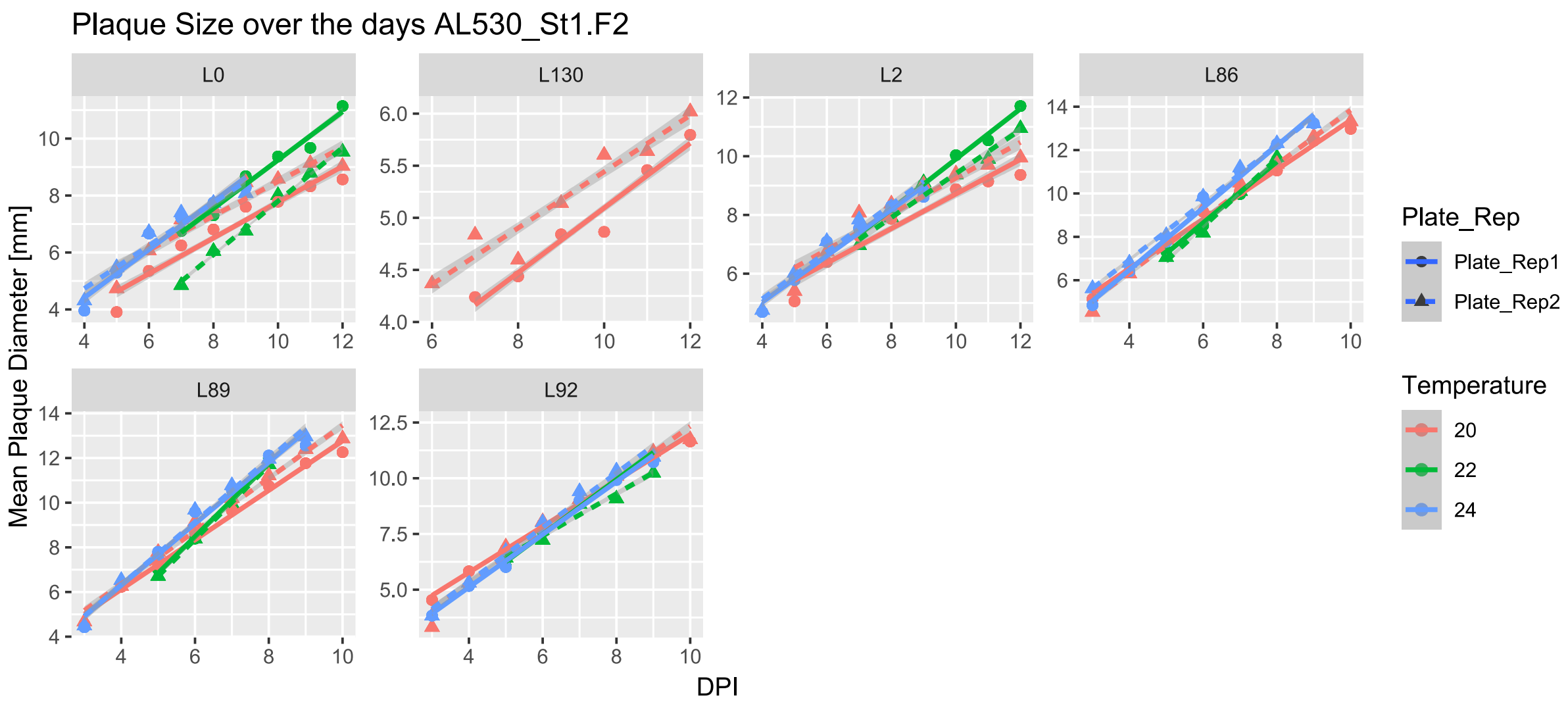

Figure S16 Slope fits of spot-plaque increase rates across days for infections for warmed treatments of host AL530_St1.F2 . Shaded areas indicate standard errors from linear model fits. Shape indicates plaque replicate. Colors indicate the different temperatures.

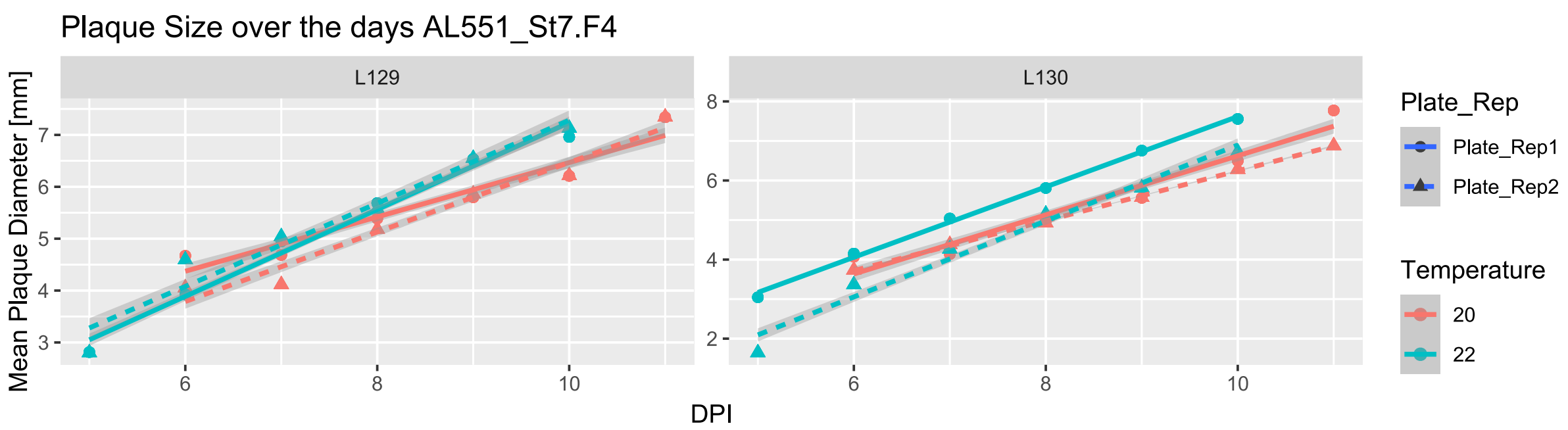

Figure S17 Slope fits of spot-plaque increase rates across days for infections for warmed treatments of host AL551_St7.F4. Shaded areas indicate standard errors from linear model fits. Shape indicates plaque replicate. Colors indicate the different temperatures.

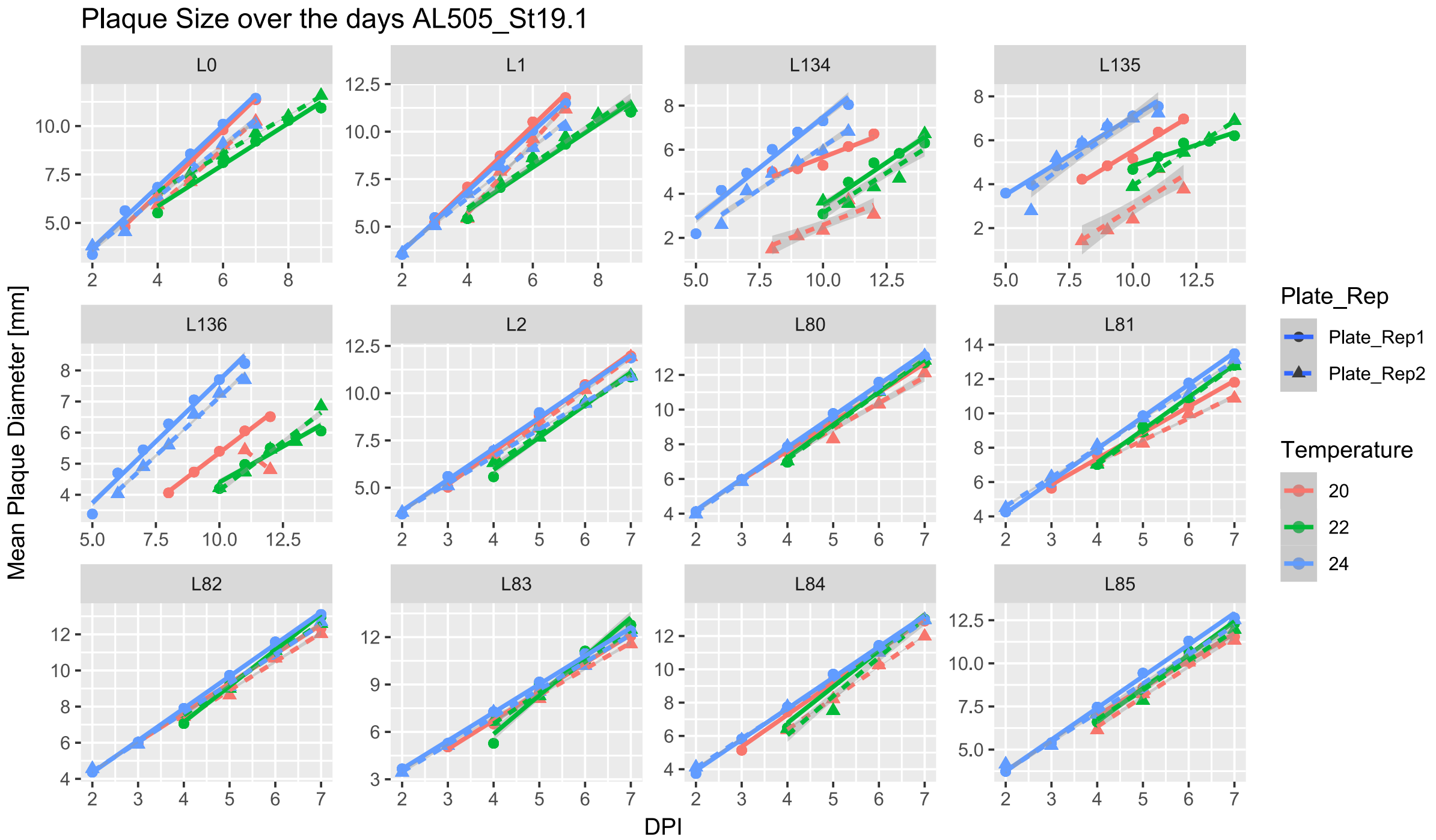

Figure S18 Slope fits of spot-plaque increase rates across days for infections for warmed treatments of host AL505_St19.1. Shaded areas indicate standard errors from linear model fits. Shape indicates plaque replicate. Colors indicate the different temperatures

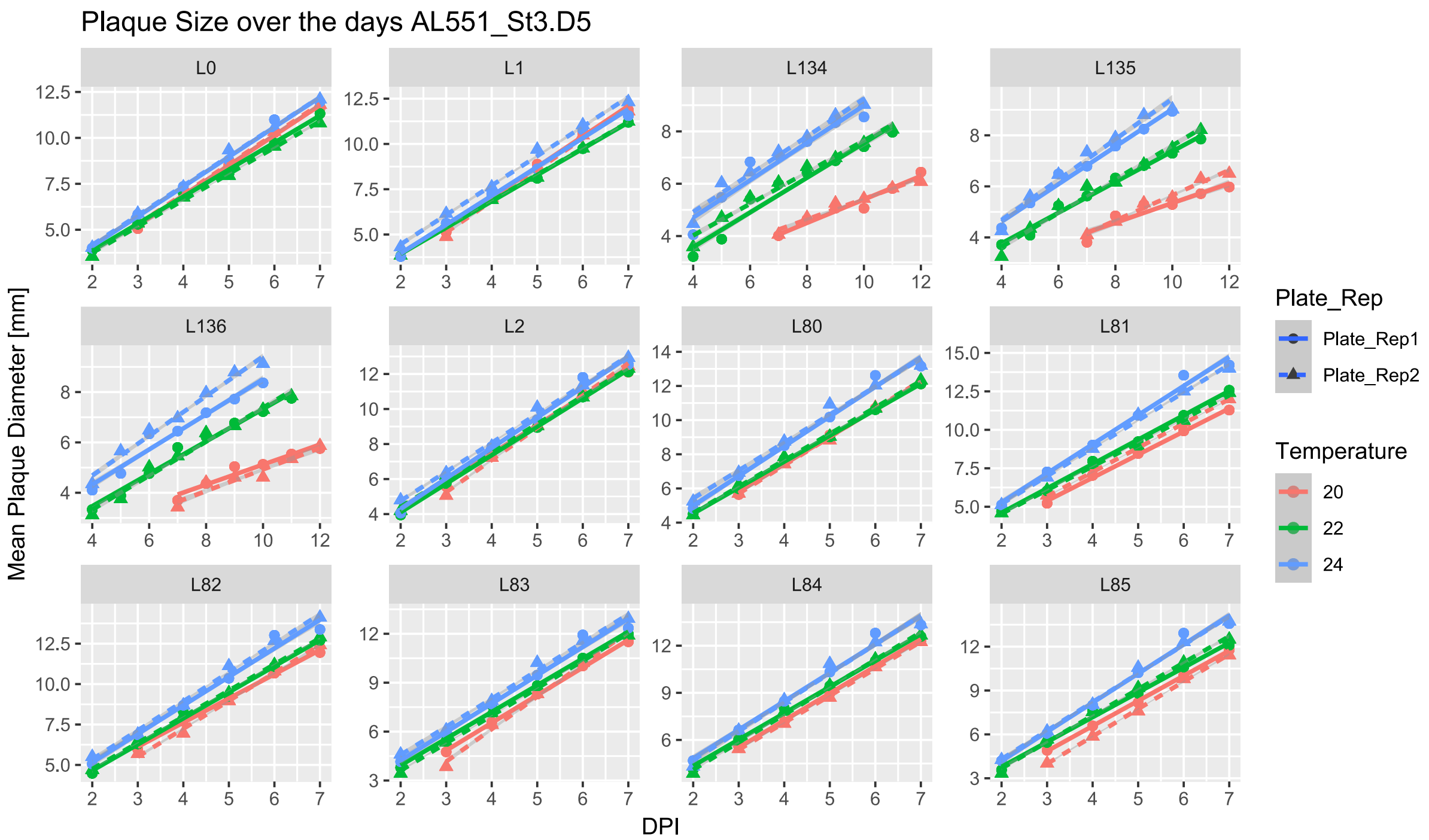

Figure S19 Slope fits of spot-plaque increase rates across days for infections for warmed treatments of host AL551_St3.D5. Shaded areas indicate standard errors from linear model fits. Shape indicates plaque replicate. Colors indicate the different temperatures

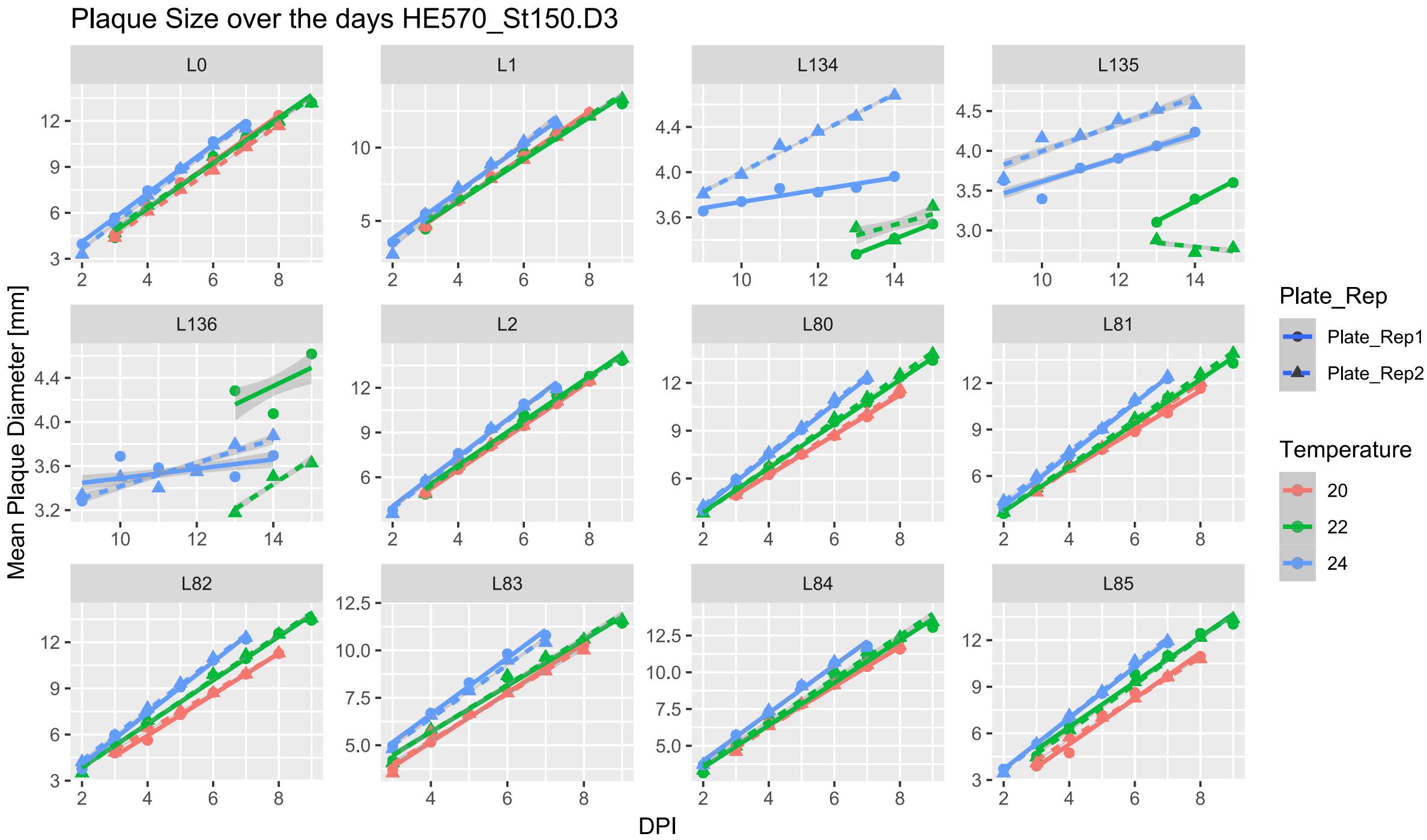

Figure S20 Slope fits of spot-plaque increase rates across days for infections for warmed treatments of host HE570_St150.D3. Shaded areas indicate standard errors from linear model fits. Shape indicates plaque replicate. Colors indicate the different temperatures

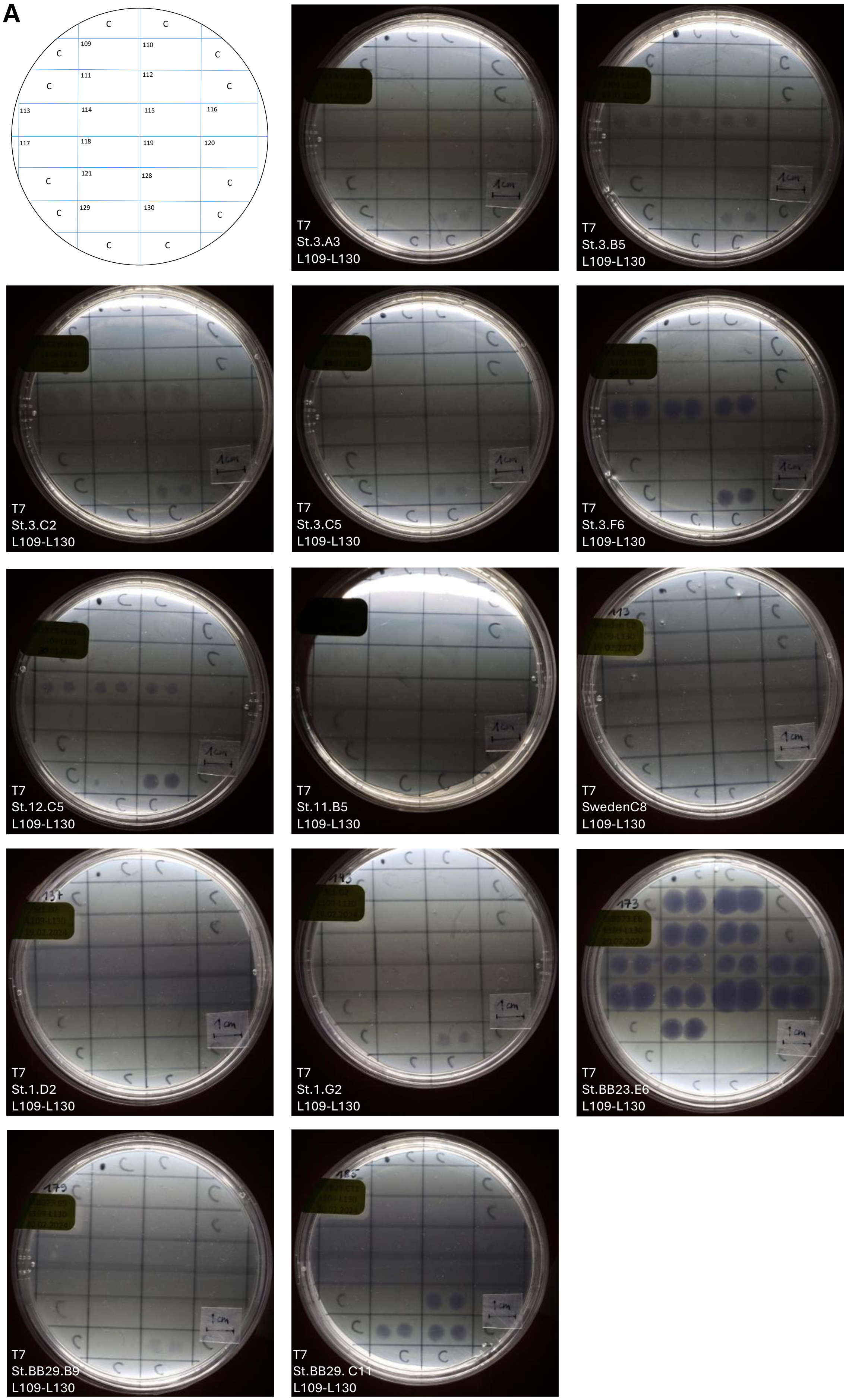
A
C
C
109
110
C
C
111
112
C
C
113
114
115
116
117
118
119
120
C
121
128
C
C
129
130
C
C
C
T7
St.3.A3
L109-L130
1cm
T7
St.3.B5
L109-L130
1cm
T7
St.3.C2
L109-L130
1cm
T7
St.3.C5
L109-L130
1cm
T7
St.3.F6
L109-L130
1cm
T7
St.12.C5
L109-L130
1cm
T7
St.11.B5
L109-L130
1cm
T7
SwedenC8
L109-L130
1cm
T7
St.1.D2
L109-L130
1cm
T7
St.1.G2
L109-L130
1cm
T7
St.BB23.E6
L109-L130
1cm
T7
St.BB29.B9
L109-L130
1cm
T7
St.BB29. C11
L109-L130
1cm

B

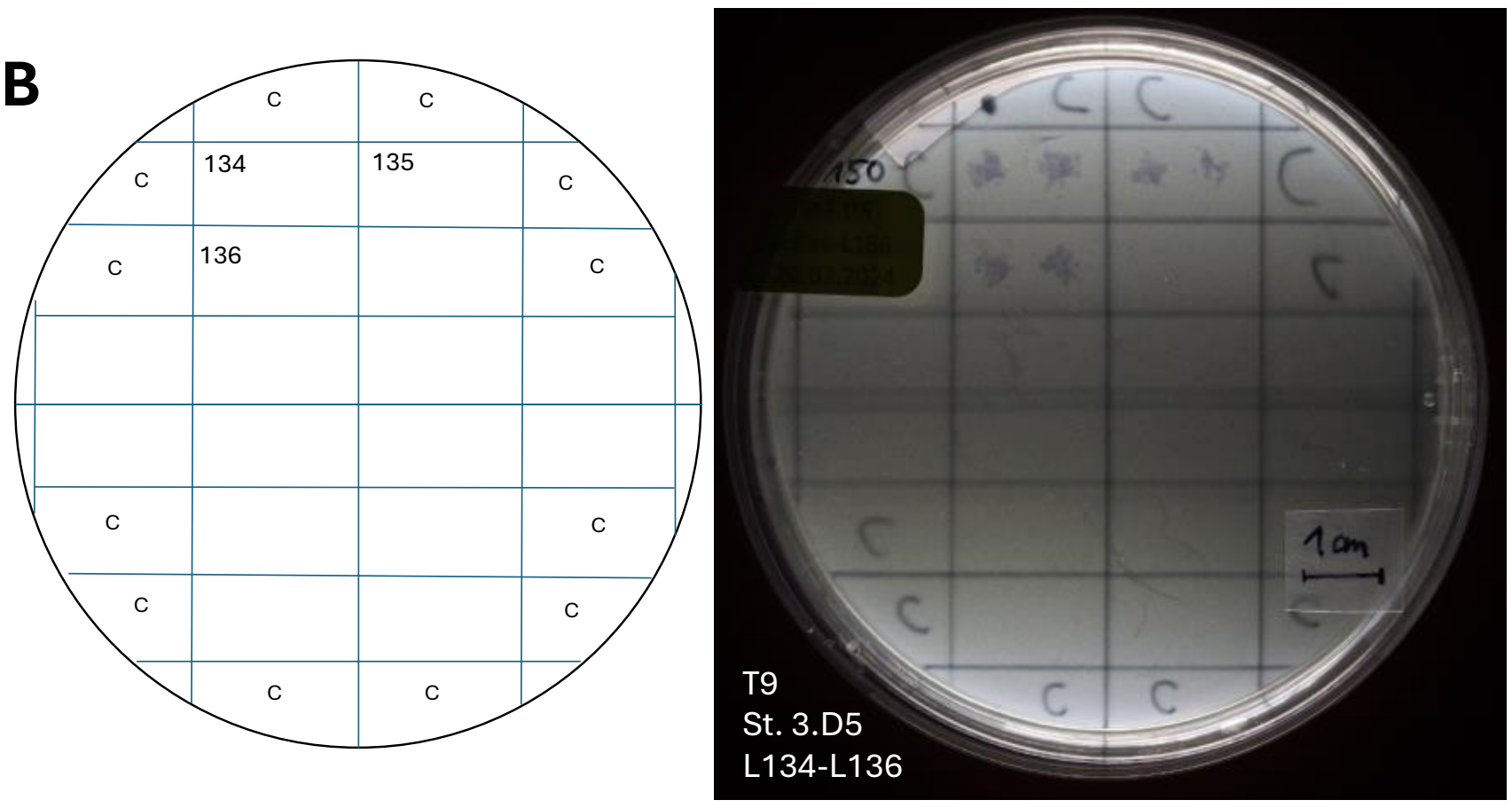

Figure S21 A Example of agar plates from big cross infection (18°C) showing different plates of infected hosts seven days after infection for the lysates L109-L130. The schematic in the upper left corner shows where which viruses are plated. The photos show that viruses L113-L115 as well as L130 often show faint clearing of spot-plaques. **B** Example of agar plate from big cross infection (18°C) showing the spotted plaque formation of lysates L113-L115 on host AL551_St3.D5 nine days after infection.

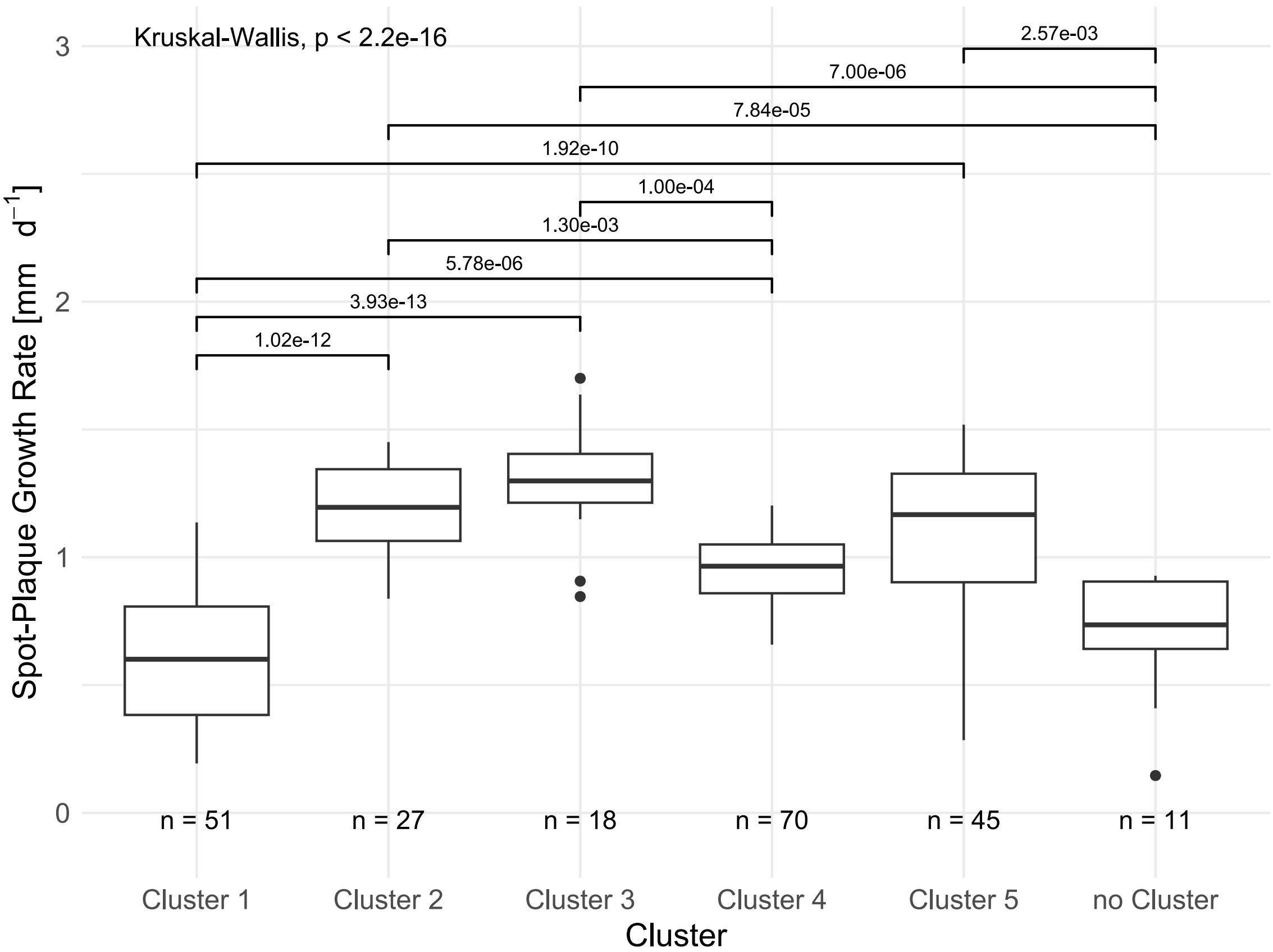

Figure S22 Comparison of spot-plaque rates between clusters. The Kruskal-Wallis test was used to test for statistical differences in rates between the clusters (p-value<2.2e-16). No Cluster refers to infections that were not grouped into the clusters in the network analysis. Bars with significance levels show results of Dunn test post-hoc pairwise comparisons between the different clusters. The boxes of

the boxplots represent the 25th to 75th percentile of the data with the line within the box representing the median (50th percentile), the whiskers include the smallest and largest values without the outliers (outside 1.5 × IQR; shown as points).

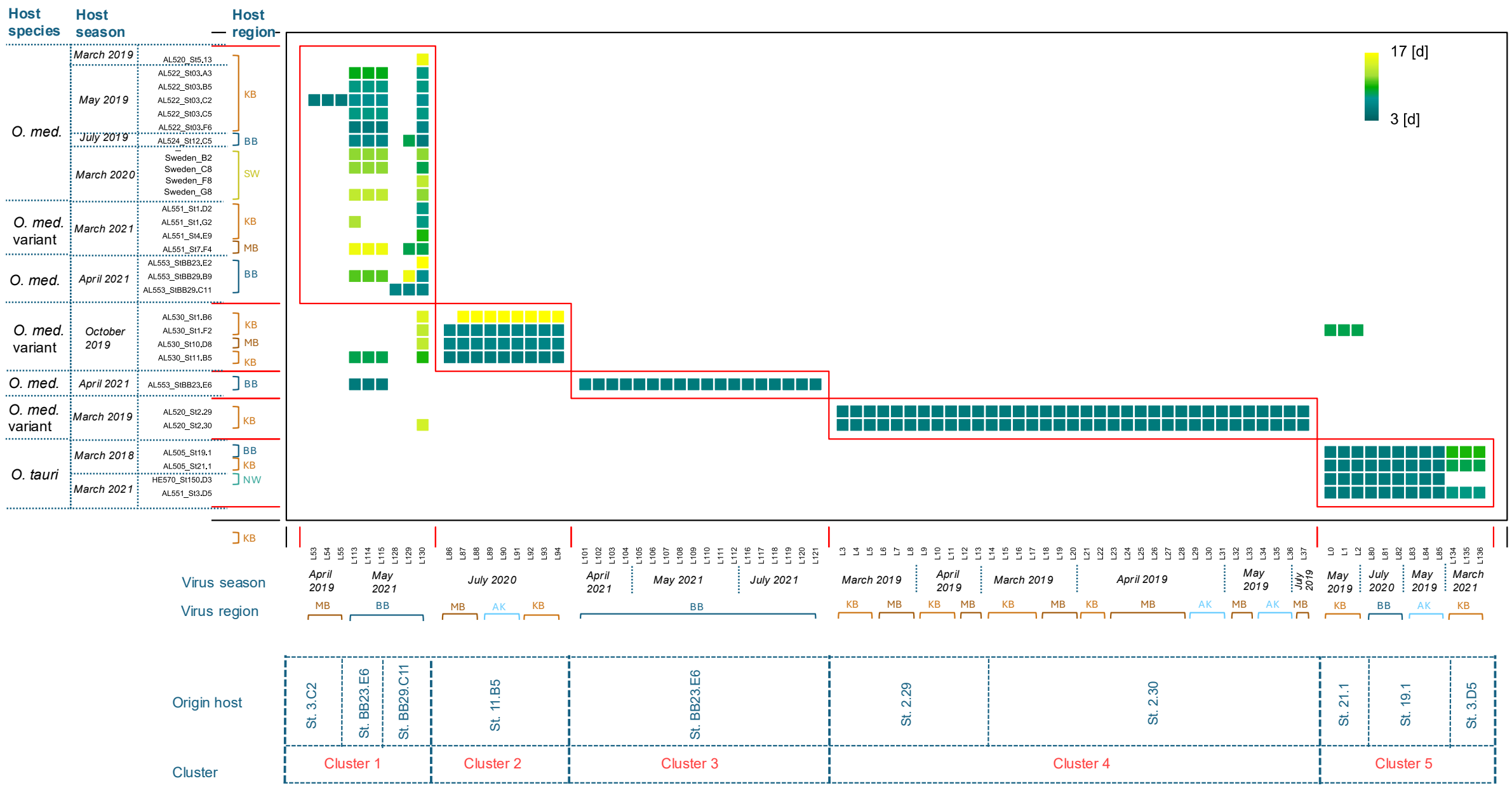

Figure S23 Infection matrix with first visible day of infection (DoI) as overlay. DoI varied between 3 and 17 days, but (with a few exceptions) is mostly homogenous for clusters two to five and, opposing to that, shows high variation in cluster one. The x-axis shows viruses with their respective information about origin hosts, regional and seasonal origin and cluster names. The y-axis shows hosts with their species designation, seasonal and regional origin.

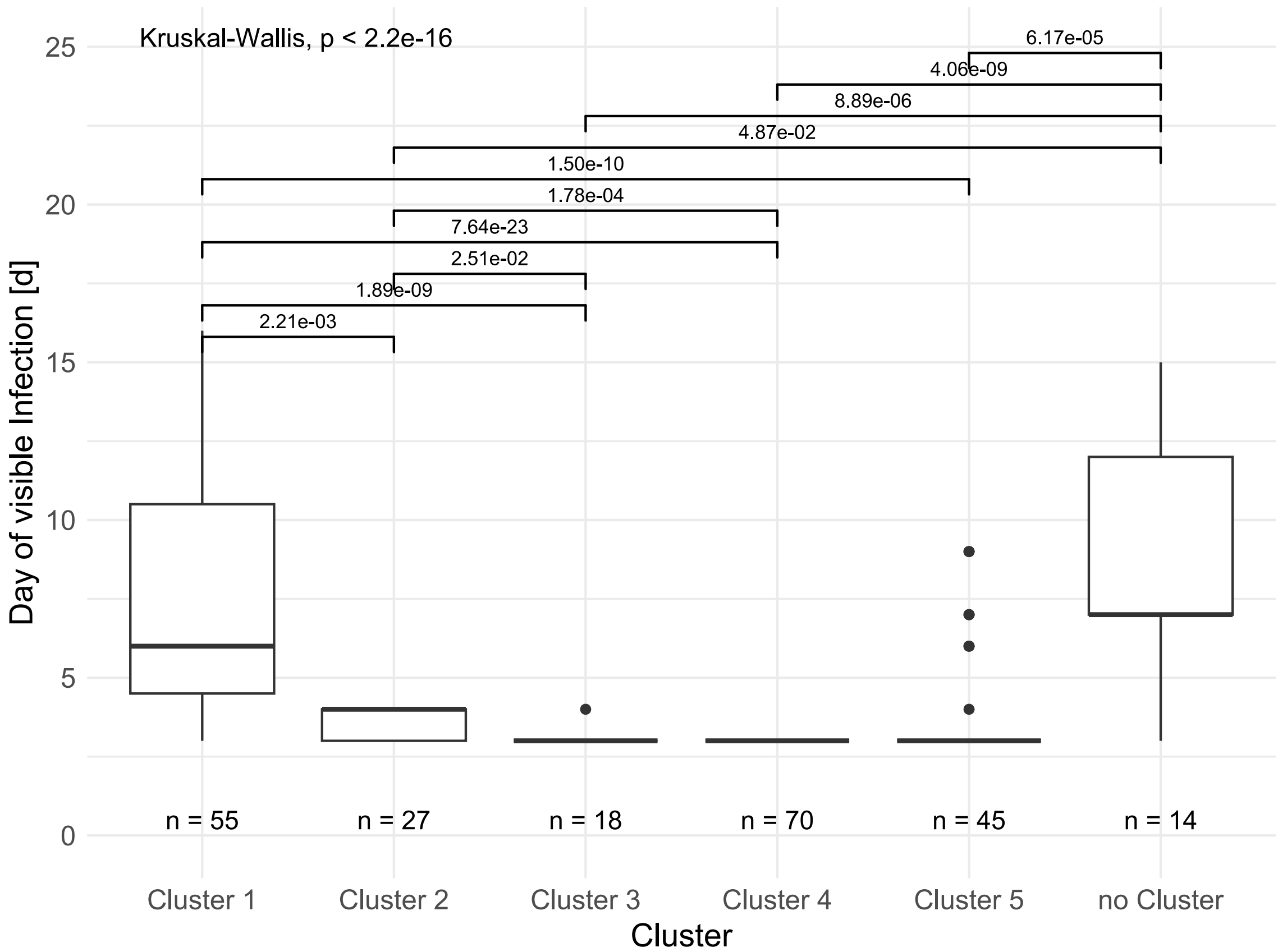

Figure S24 First visible day of infection (DoI) between the clusters. Statistical significance of differences was tested using the Kruskal-Wallis rank sum test ($p < 2.2e16$). No Cluster refers to infections that were not grouped into the clusters in the network analysis. Bars with significance levels between the different boxes show results of Dunn test pairwise comparisons between the different clusters. The boxes of the boxplots represent the 25th to 75th percentile of the data with the line within the box representing the median (50th percentile), the whiskers include the smallest and largest values without the outliers (outside 1.5 × IQR; shown as points).

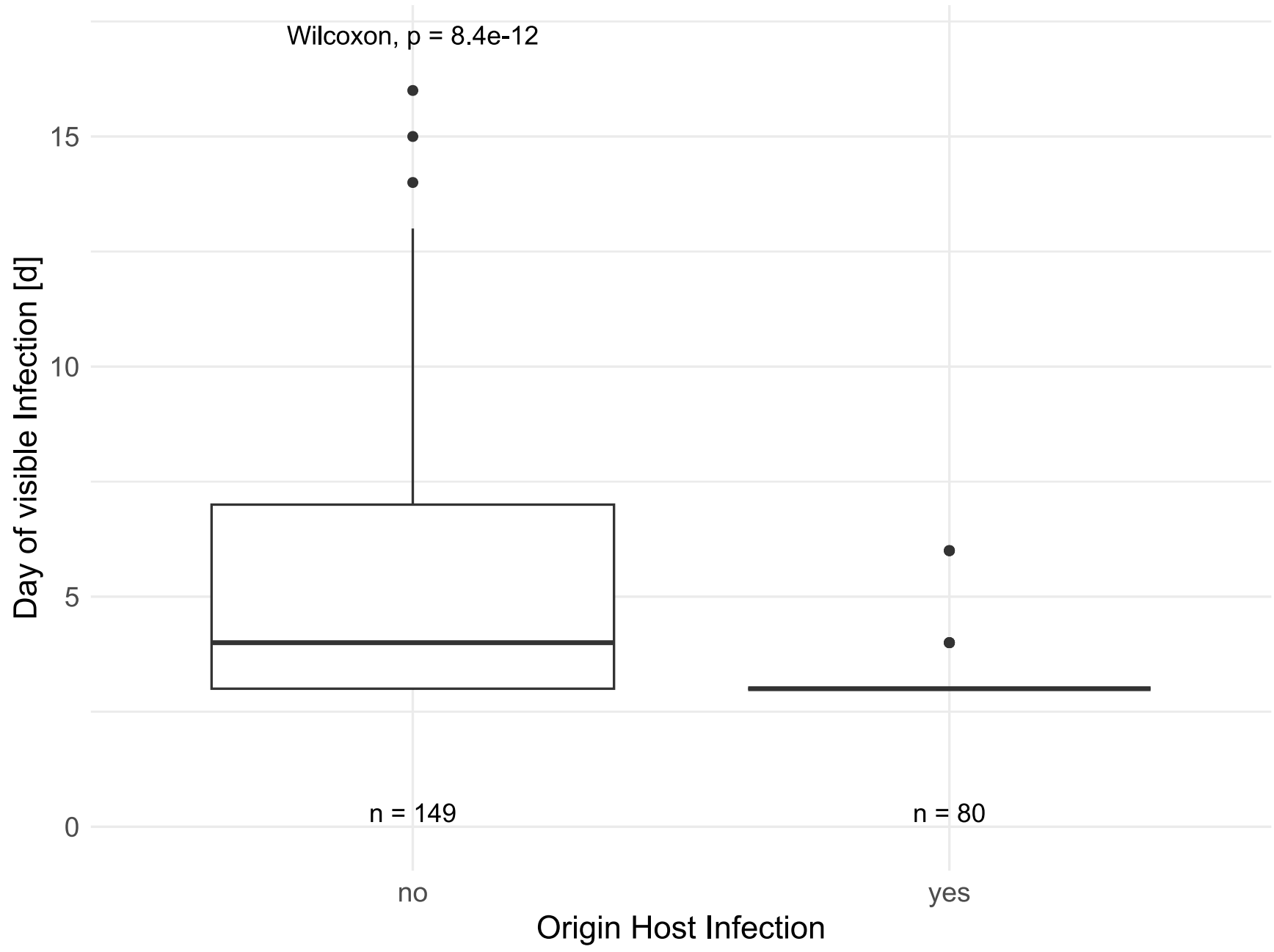

Figure S25 Comparison of first day of visible infection (DoI9 between viruses that infected their own host (yes), meaning the host they were isolated from or a foreign host (no). A Wilcoxon rank sum test was performed to test for statistical differences in rates (p-value=8.4e-12). The boxes of the boxplots represent the 25th to 75th percentile of the data with the line within the box representing the median (50th percentile), the whiskers include the smallest and largest values without the outliers (outside 1.5 × IQR; shown as points).

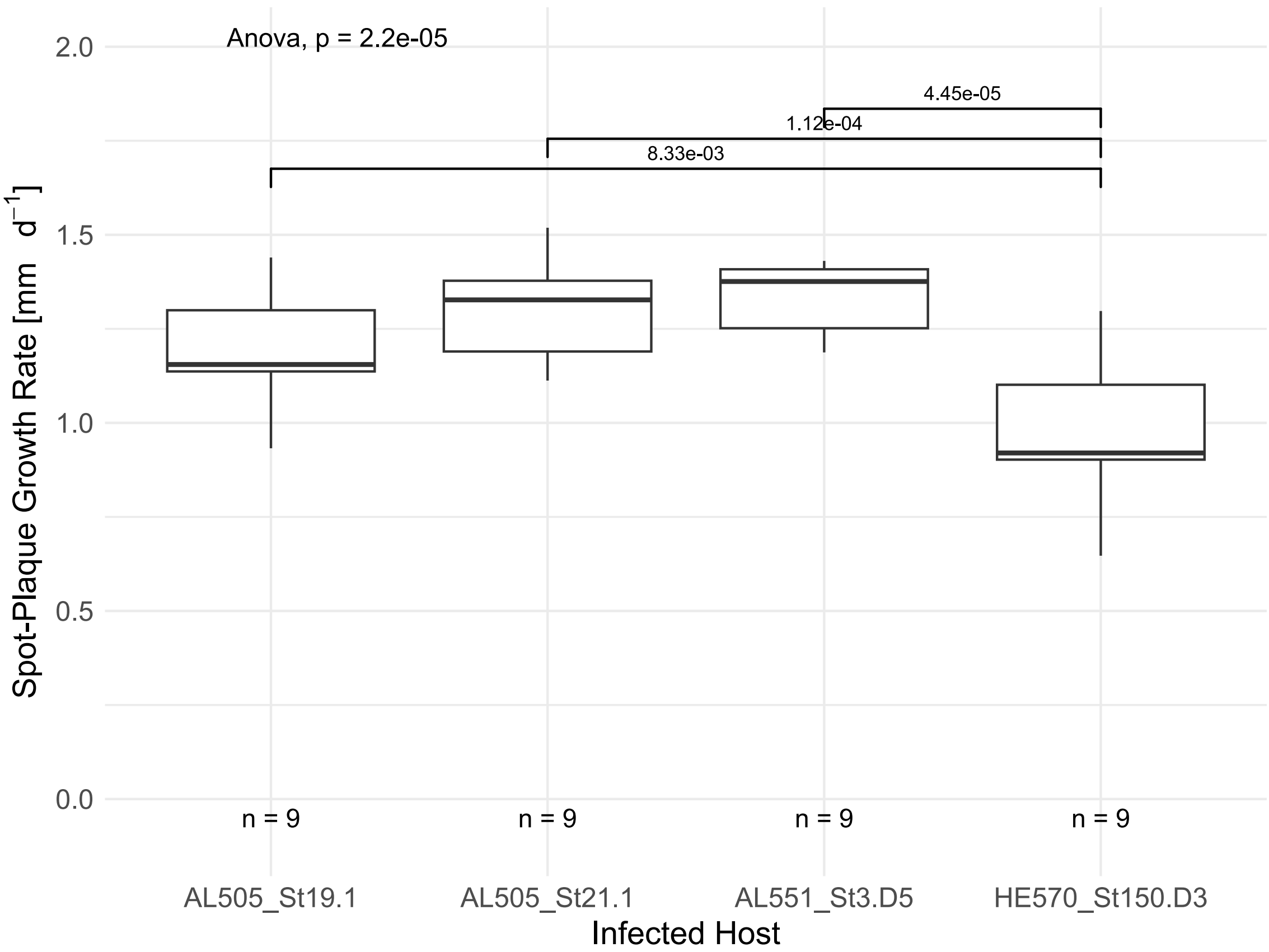

Figure S26 Spot-plaque growth rates of viruses that infected all *O. tauri* hosts (therefore excluding viruses L134-L136) between the different infected *O. tauri* hosts. An ANOVA was performed to test for statistical differences in rates (p-value=2.2e-05), followed by a Tukey's HSD post-hoc test to do pairwise comparisons, illustrated through the bars between boxplots with annotated significance levels. The boxes of the boxplots represent the 25th to 75th percentile of the data with the line within the box representing the median (50th percentile), the whiskers include the smallest and largest values without the outliers (outside 1.5 × IQR; shown as points).

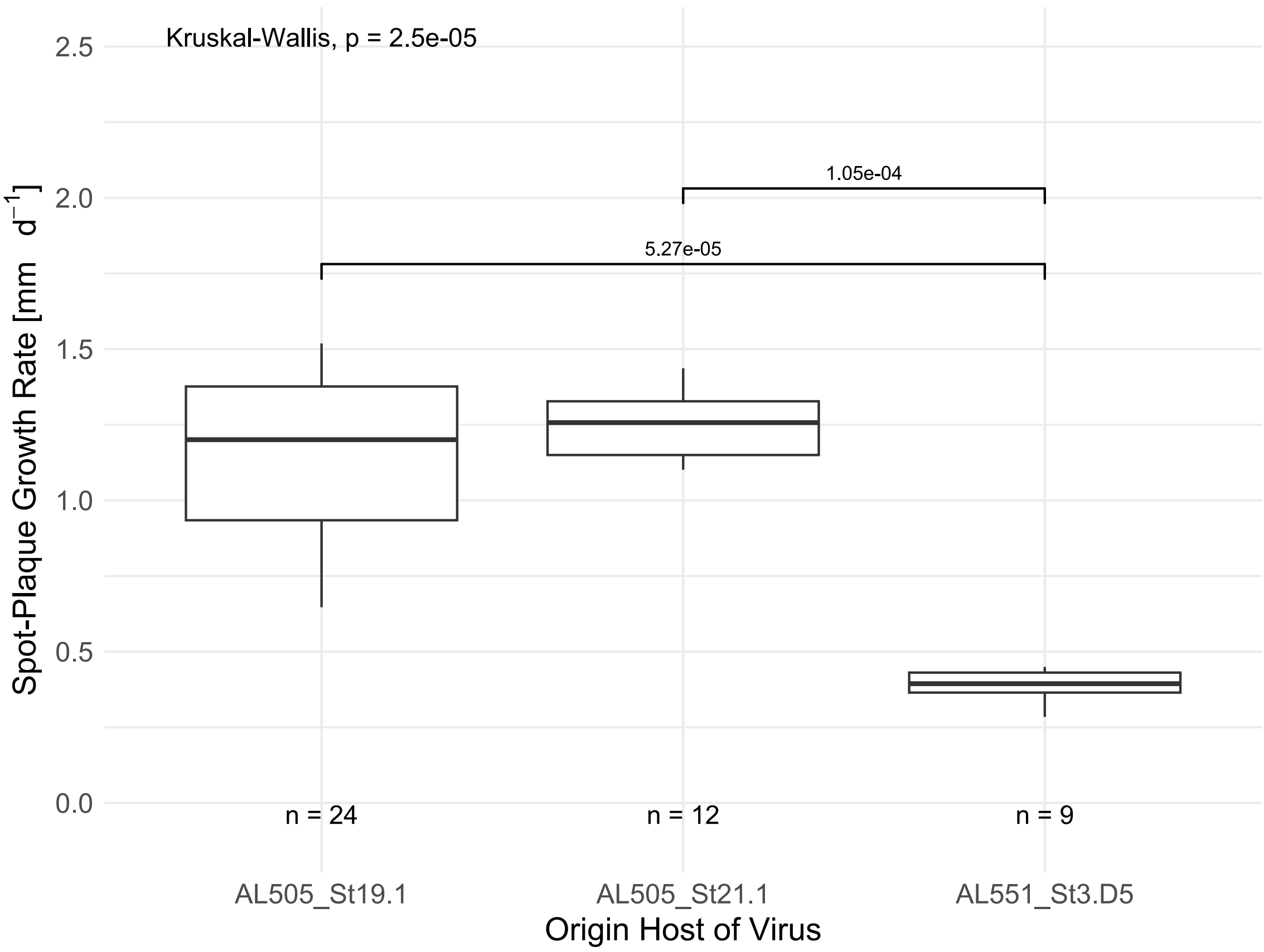

Figure S27 Comparison of spot-plaque growth rates of *O. tauri* infections in Cluster five between the viruses of different origin hosts. The Kruskal-Wallis test was used to test for statistical differences in rates between the viruses of different origin hosts (p-value = 2.5e-05). Bars with significance levels show results of Dunn test post-hoc pairwise comparisons between the different clusters. The boxes of the boxplots represent the 25th to 75th percentile of the data with the line within the box representing the median (50th percentile), the whiskers include the smallest and largest values without the outliers (outside 1.5 × IQR; shown as points).

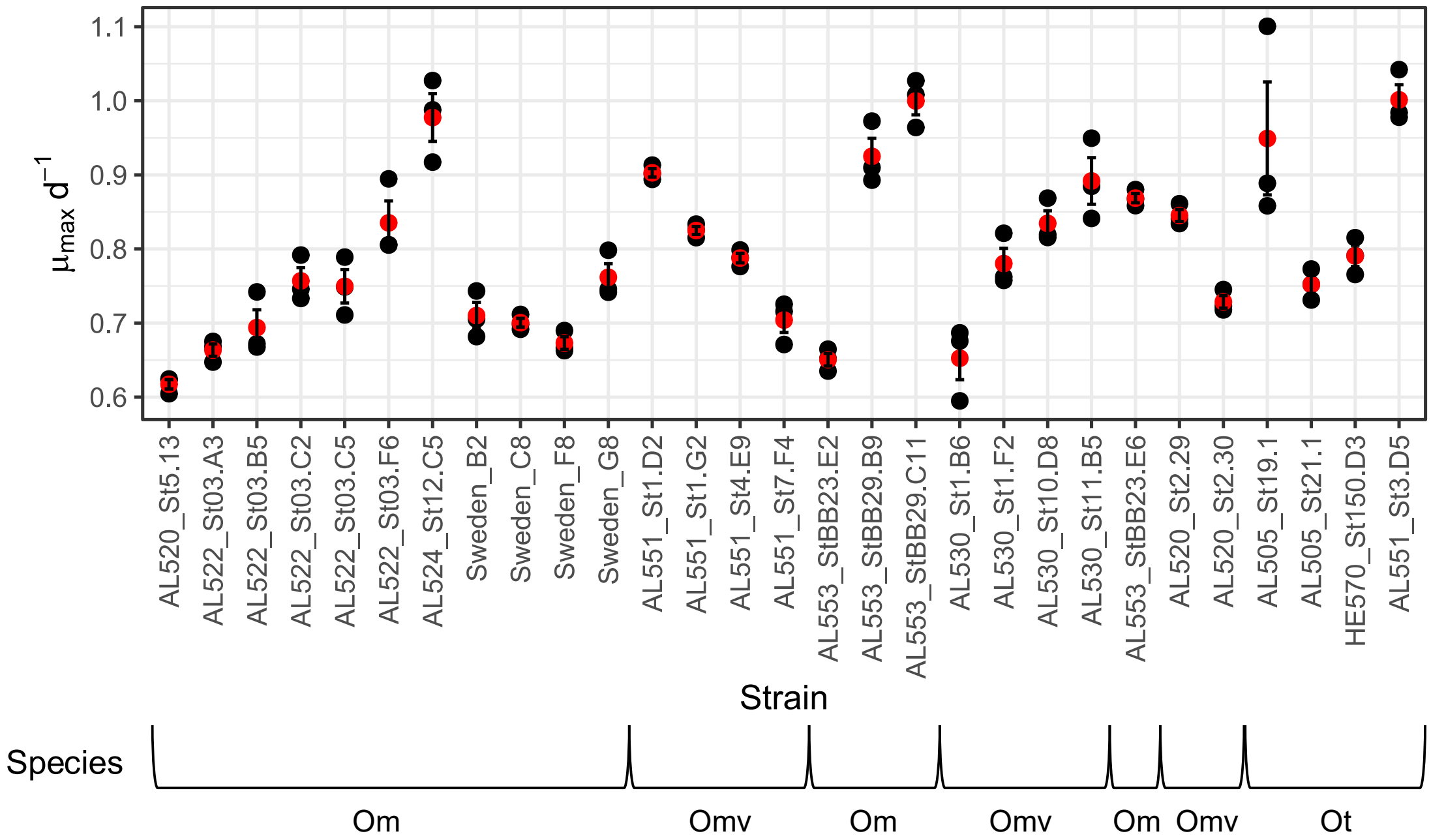

Figure S28 Maximum growth rates (d-1) of hosts at 18°C (without viruses), order as seen in the network analysis in Figure 1 of the main manuscript. Red points indicate mean with standard error bars. For species annotation, Om = *Ostreococcus mediterraneus*, Omv = *Ostreococcus mediterraneus* variant, Ot= *Ostreococcus tauri.* Data was collected in Peters et al. (2026).

Supplementary Tables

Table S1 Overview of the *Ostreococcus* strains as well as their origin region and time, species and location isolation coordinates. Strain names marked with an asterisk refer to strains isolated in Listmann et al. (2021) and Listmann et al. (2023), the remaining strains are the newer isolates.

Strain	Region	Isolation Time	Species	Latitude	Longitude
AL505_St19.1*	Bornholm	March_2018	*O. tauri*	55.6119444	15.3038889
AL505_St21.1*	Kiel	March_2018	*O. tauri*	54.5241667	11.3266667
AL520_St5.13*	Kiel	March_2019	*O. mediterraneus*	54.6361111	11.0036111
AL520_St2.29*	Kiel	March_2019	Hybrid	54.3775	10.9136
AL520_St2.30*	Kiel	March_2019	Hybrid	54.3775	10.9136
AL522_St03.A3*	Kiel	May_2019	*O. mediterraneus*	54.1986111	11.4688889
AL522_St03.B5*	Kiel	May_2019	*O. mediterraneus*	54.1986111	11.4688889
AL522_St03.C2*	Kiel	May_2019	*O. mediterraneus*	54.1986111	11.4688889
AL522_St03.C5*	Kiel	May_2019	*O. mediterraneus*	54.1986111	11.4688889
AL522_St03.F6*	Kiel	May_2019	*O. mediterraneus*	54.1986111	11.4688889
AL524_St12.C5*	Bornholm	July_2019	*O. mediterraneus*	55.0502778	13.6666667
AL530_St1.B4*	Kiel	October_2019	Hybrid	54.6111111	10.3008333
AL530_St1.B6*	Kiel	October_2019	Hybrid	54.6111111	10.3008333
AL530_St1.F2*	Kiel	October_2019	Hybrid	54.6111111	10.3008333
AL530_St10.D8*	Mecklenburg	October_2019	Hybrid	54.3011111	11.3822222
AL530_St11.B5*	Kiel	October_2019	Hybrid	54.6727778	11.0347222
Sweden_B2*	Skagerrak	March_2020	*O. mediterraneus*	58.879518	11.124977
Sweden_B5*	Skagerrak	March_2020	*O. mediterraneus*	58.879518	11.124977
Sweden_C8*	Skagerrak	March_2020	*O. mediterraneus*	58.879518	11.124977
Sweden_F8*	Skagerrak	March_2020	*O. mediterraneus*	58.879518	11.124977
Sweden_G8*	Skagerrak	March_2020	*O. mediterraneus*	58.879518	11.124977
HE570_St150.D3	Norway	March_2021	*O. tauri*	60.692	5.158
AL551_St1.D2	Kiel	March_2021	Hybrid	54.7275	10.0797222
AL551_St1.G2	Kiel	March_2021	Hybrid	54.7275	10.0797222
AL551_St3.D5	Kiel	March_2021	*O. tauri*	54.89	10.3566667
AL551_St4.E9	Kiel	March_2021	Hybrid	54.4894444	10.5916667
AL551_St7.F4	Mecklenburg	March_2021	Hybrid	54.4016667	11.8330556
AL553_StBB23.E2	Bornholm	April_2021	*O. mediterraneus*	55.2969444	15.7508333
AL553_StBB23.E6	Bornholm	April_2021	*O. mediterraneus*	55.2969444	15.7508333
AL553_StBB29.B9	Bornholm	April_2021	*O. mediterraneus*	55.1297222	16.0075
AL553_StBB29.C11	Bornholm	April_2021	*O. mediterraneus*	55.1297222	16.0075

Table S2 Overview of the *Ostreococcus* viruses as well as their origin host, region and time. For region abbreviations, a different number in front of the region means a different station. KB= Kiel Bight, MB=Mecklenburg Bight. AK=Arkona Basin, BB=Bornholm Basin.

Virus ID-Number	Origin Host of Virus	Virus Isolation Region	Virus Isolation Time
L0	AL505_St21.1	02_KB	May 2019
L1	AL505_St21.1	02_KB	May 2019
L2	AL505_St21.1	02_KB	May 2019
L3	AL520_St2.29	02_KB	March 2019
L4	AL520_St2.29	02_KB	March 2019
L5	AL520_St2.29	02_KB	March 2019
L6	AL520_St2.29	03_MB	March 2019
L7	AL520_St2.29	03_MB	March 2019
L8	AL520_St2.29	03_MB	March 2019
L9	AL520_St2.29	02_KB	April 2019
L10	AL520_St2.29	02_KB	April 2019
L11	AL520_St2.29	02_KB	April 2019
L12	AL520_St2.29	03_MB	April 2019
L13	AL520_St2.29	03_MB	April 2019
L14	AL520_St2.30	02_KB	March 2019
L15	AL520_St2.30	02_KB	March 2019
L16	AL520_St2.30	02_KB	March 2019
L17	AL520_St2.30	02_KB	March 2019
L18	AL520_St2.30	03_MB	March 2019
L19	AL520_St2.30	03_MB	March 2019
L20	AL520_St2.30	03_MB	March 2019
L21	AL520_St2.30	02_KB	April 2019
L22	AL520_St2.30	02_KB	April 2019
L23	AL520_St2.30	03_MB	April 2019
L24	AL520_St2.30	03_MB	April 2019
L25	AL520_St2.30	03_MB	April 2019
L26	AL520_St2.30	03_MB	April 2019
L27	AL520_St2.30	03_MB	April 2019
L28	AL520_St2.30	03_MB	April 2019
L29	AL520_St2.30	04_AK	April 2019
L30	AL520_St2.30	04_AK	April 2019
L31	AL520_St2.30	04_AK	April 2019
L32	AL520_St2.30	03_MB	May 2019
L33	AL520_St2.30	03_MB	May 2019
L34	AL520_St2.30	04_AK	May 2019
L35	AL520_St2.30	04_AK	May 2019
L36	AL520_St2.30	04_AK	May 2019
L37	AL520_St2.30	03_MB	July 2019
L53	AL522_St3.C2	03_MB	April 2019
L54	AL522_St3.C2	03_MB	April 2019
L55	AL522_St3.C2	03_MB	April 2019
L80	AL505_St19.1	05_BB	July 2020
L81	AL505_St19.1	05_BB	July 2020
L82	AL505_St19.1	05_BB	July 2020
L83	AL505_St19.1	04_AK	May 2019
L84	AL505_St19.1	04_AK	May 2019
L85	AL505_St19.1	04_AK	May 2019
L86	AL530_St11.B5	03_MB	July 2020
L87	AL530_St11.B5	03_MB	July 2020

L88	AL530_St11.B5	03_MB	July 2020
L89	AL530_St11.B5	04_AK	July 2020
L90	AL530_St11.B5	04_AK	July 2020
L91	AL530_St11.B5	04_AK	July 2020
L92	AL530_St11.B5	02_KB	July 2020
L93	AL530_St11.B5	02_KB	July 2020
L94	AL530_St11.B5	02_KB	July 2020
L101	AL553_StBB23_E6	05_BB	April 2021
L102	AL553_StBB23_E6	05_BB	April 2021
L103	AL553_StBB23_E6	05_BB	April 2021
L104	AL553_StBB23_E6	05_BB	April 2021
L105	AL553_StBB23_E6	05_BB	May 2021
L106	AL553_StBB23_E6	05_BB	May 2021
L107	AL553_StBB23_E6	05_BB	May 2021
L108	AL553_StBB23_E6	05_BB	May 2021
L109	AL553_StBB23_E6	05_BB	May 2021
L110	AL553_StBB23_E6	05_BB	May 2021
L111	AL553_StBB23_E6	05_BB	May 2021
L112	AL553_StBB23_E6	05_BB	May 2021
L113	AL553_StBB23_E6	05_BB	May 2021
L114	AL553_StBB23_E6	05_BB	May 2021
L115	AL553_StBB23_E6	05_BB	May 2021
L116	AL553_StBB23_E6	05_BB	July 2021
L117	AL553_StBB23_E6	05_BB	July 2021
L118	AL553_StBB23_E6	05_BB	July 2021
L119	AL553_StBB23_E6	05_BB	July 2021
L120	AL553_StBB23_E6	05_BB	July 2021
L121	AL553_StBB23_E6	05_BB	July 2021
L128	AL553_StBB29_C11	05_BB	May 2021
L129	AL553_StBB29_C11	05_BB	May 2021
L130	AL553_StBB29_C11	05_BB	May 2021
L134	AL551_St03_D5	02_KB	July 2021
L135	AL551_St03_D5	02_KB	July 2021
L136	AL551_St03_D5	02_KB	July 2021

Supplementary Methods

Detailed Methods Preparation and storage of Agar Plates

Plates were incubated at 18 °C under a 12:12 h light:dark cycle (~50–150 µmol m^{-2} s^{-1}) and inspected daily for spot-plaque formation. Plate positions were rotated every second day to account for the light variation. Photographs were taken (see Supplementary Figure S10 for photo set up) beginning on day 3 post-infection (2 days post infection for warming cross infection) or on the day spot-plaques first became visible, and subsequently every day until plaques could no longer be reliably measured (e.g. culture decline, spot-plaques touching). All images were captured at the same time of day (±30 min). Spot-plaque diameter was measured in ImageJ (horizontally, vertically, diagonally) and averaged to account for spot-plaques that did not grow perfectly round. In addition, the day of first visible infection was tracked (DoI).

Cross Infections under Warming

The strain AL551_St.7.F4 strain did not survive acclimation at 24°C, thus, this temperature treatment was omitted for that host. Following plating in molten agar, cells exhibited transient depigmentation and reduced apparent viability at all warmed temperatures for strain AL522_St03.C2 and at 22°C for strain AL520_St2.29. Although for some plates surviving cells were partly able to recover and proliferate and viral infection was observable, regrowth was spatially heterogeneous, forming patchy rather than uniform lawns; these plates were therefore excluded to maintain consistent experimental conditions.

References

Listmann, Luisa; Kerl, Franziska; Martens, Nele; Schaum, C-Elisa (2021): Differences in Carbon Acquisition Could Explain Adaptive Responses in a Baltic Sea Pico-Phytoplankton. In: *Front. Mar. Sci.* 8, Artikel 740763. DOI: 10.3389/fmars.2021.740763.

Listmann, Luisa; Peters, Carina; Rahlff, Janina; Esser, Sarah P.; Schaum, C-Elisa (2023): Seasonality and Strain Specificity Drive Rapid Co-evolution in an Ostreococcus-Virus System from the Western Baltic Sea. In: *Microbial ecology* 86 (4), S. 2414–2423. DOI: 10.1007/s00248-023-02243-5.

Peters, Carina; Vogel, Marie; Listmann, Luisa; Schaum, C.-Elisa (2026): Thermal responses of Baltic Sea Ostreococcus vary in their levels of intra-and interspecific variability. bioRxiv [Preprint] (2026). 10.64898/2026.01.13.698997 (accessed February 2nd 2026). DOI: 10.64898/2026.01.13.698997.
